# High-throughput sequencing of 16S rRNA Gene Reveals Substantial Bacterial Diversity on the Municipal Dumpsite

**DOI:** 10.1186/s12866-016-0758-8

**Published:** 2016-07-11

**Authors:** Kilaza Samson Mwaikono, Solomon Maina, Aswathy Sebastian, Megan Schilling, Vivek Kapur, Paul Gwakisa

**Affiliations:** Department of Science and Laboratory Technology, Dar es Salaam Institute of Technology, Dar es Salaam, Tanzania; School of Life Sciences and Bioengineering, The Nelson Mandela African Institution of Science and Technology, P.O. Box 447, Arusha, Tanzania; BecA-ILRI Hub International Livestock Research Institute, P. O. Box 30709, Nairobi, Kenya; Genome Sciences Centre, Faculty of Veterinary Medicine, Sokoine University of Agriculture, Morogoro, Tanzania; Departments of Biochemistry and Molecular Biology, W238A Millennium Science Complex, Penn State University, University Park, PA 16802 USA; Huck Institutes of Life Sciences, Molecular Cellular and Integrative Biosciences, the Pennsylvania State University, 204 Wartik Laboratories, University Park, PA 16802 USA

**Keywords:** Municipal dumpsite, Solid waste, Molecular diversity, 16S rRNA, Illumina MiSeq

## Abstract

**Background:**

Multiple types of solid waste in developing countries is disposed of together in dumpsites where there is interaction between humans, animals and the bacteria in the waste. To study the bacteria at the dumpsite and the associated risks, previous studies have focused on culturable, leaving behind a great number of unculturable bacteria. This study focuses on a more comprehensive approach to study bacteria at the dumpsite. Since the site comprised of unsorted wastes, a qualitative survey was first performed to identify the variety of solid waste as this has influence on the microbial composition. Thus, domestic (Dom), biomedical (Biom), river sludge (Riv), and fecal material of pigs scavenging on the dumpsite (FecD) were sampled. Total DNA was extracted from 78 samples and the v4-16S rRNA amplicons was characterized using an Illumina MiSeq platform.

**Results:**

A total of 8,469,294 sequences passed quality control. Catchall analysis predicted a mean of 8243 species per sample. Diversity was high with an average InvSimpson index of 44.21 ± 1.44. A total of 35 phyla were detected and the predominant were *Firmicutes* (38 %), *Proteobacteri*a (35 %), *Bacteroidetes* (13 %) and *Actinobacteria* (3 %). Overall 76,862 OTUs were detected, however, only 20 % were found more than 10 times. The predominant OTUs were *Acinetobacter* (12.1 %), *Clostridium sensu stricto* (4.8 %), *Proteinclasticum* and *Lactobacillus* both at (3.4 %), *Enterococcus (*2.9 %) and *Escherichia/Shigella* (1.7 %). Indicator analysis (*P* ≤ 0.05, indicator value ≥ 70) shows that *Halomonas, Idiomarina, Tisierella* and *Proteiniclasticum* were associated with Biom; *Enterococcus, Bifidobacteria*, and *Clostridium sensu stricto* with FecD and *Flavobacteria, Lysobacter* and *Commamonas* to Riv. *Acinetobacter* and *Clostridium sensu stricto* were found in 62 % and 49 % of all samples, respectively, at the relative abundance of 1 %. None of OTUs was found across all samples.

**Conclusions:**

This study provides a comprehensive report on the abundance and diversity bacteria in municipal dumpsite. The species richness reported here shows the complexity of this man-made ecosystem and calls for further research to assess for a link between human diseases and the dumpsite. This would provide insight into proper disposal of the waste, as well as, limit the risks to human health associated with the dumpsite.

**Electronic supplementary material:**

The online version of this article (doi:10.1186/s12866-016-0758-8) contains supplementary material, which is available to authorized users.

## Background

The amount of solid waste generated has risen due to the increasing urban population in most developing countries [1–3]. Limited resources and inefficient infrastructure prevent proper waste separation leading to waste remaining in their production sites since it does not get transported to the final dumpsite. The lack of proper waste management systems has also created a dumpsite that includes solid waste, such as plastics, organic waste from households, markets, and abattoirs, agricultural waste, industrial waste, and chemical, pharmaceutical, and biomedical waste. Unattended wastes on dumpsites attract insects, birds, and rodents, as well as, domestic and wild animals. Livestock, such as pigs, goats, and cattle scavenge on this waste. Humans, including children also visit the dumpsite to pick waste without protective gear. The unique, man-made ecosystem in the Arusha municipality in Tanzania, representing conditions in other developing cities, has the potential to cause serious impacts to public health.

Studies on the abundance and diversity of bacteria in environments, especially at interfaces like the dumpsite with human and animal activity, would enrich our understanding of the risks from such interfaces. The presence of disease vectors, such as insects, rodents, and several other small wild animals, at dumpsites can potentially spread pathogens from the dumpsite to other habitats. This highlights the importance of a need for a comprehensive study of the abundance and diversity of bacteria in municipal dumpsites.

Previous studies have only focused on the identification of disease vectors and culturable bacteria on the dumpsite. For example, studies by Ahmed *et al.*, and Onyindo *et al.,* [4, 5] report *Periplaneta americanus* (Cockroach), *Musca domestica* (House fly), and *Ophyra leucostoma* (Black garbage fly) and *Stomoxys calcitrans* (stable fly) the most prevalent disease vectors on the dumpsite. Awisan *et al.,* [6] found *Staphylococcus aureus, Pseudomonas aerugionosa*, *Klebsiella pneumonia* and *Escherichia coli* as the aerobic and opportunistic bacteria associated to clinical diseases in Irisan dumpsite, and Emmanuel *et al.,* [7] found antibiotic resistant *Salmonella* spp., *Shigella* spp., and *Vibrio cholerae*, *Proteus* spp*.,* and *Pseudomonas* spp. from the Utisols dumpsite. However, these methods detect less than 1 % of bacteria found in a particular environment [8, 9]. Other studies have used molecular approaches to identify culturable and unculturable bacteria in a sample, yet, these techniques, such as Sanger sequencing [10, 11], are tedious and inefficient.

In the current study, total genomic DNA was extracted from samples collected from the different types of solid wastes in the dumpsites and the v4 region of the16S rRNA amplicons were sequenced using a high throughput Illumina MiSeq platform. To our knowledge, this is the first report using culture independent approaches and high throughput sequencing to study bacterial abundance and diversity in a municipal dumpsite where interaction between the microbes, animals, disease vectors, and humans is common.

## Results

### Qualitative survey of the dumpsite

Qualitative survey of the dumpsite revealed the presence of different types of unsorted solid wastes on the dumpsite. Domestic and wild feral animals as well as people were found interacting on the dumpsite. Diversity of animals and solid wastes on the dumpsite is shown in Additional file 1.

### Diversities of bacterial communities on dumpsite

A total of 8,469,294 v4 region of 16S rRNA gene sequences of bacteria from 78 solid wastes samples passed all quality control filters. The number of high quality sequences per sample ranged from 329 to 291,482 (mean 108,581, SD 56,553). Catchall analysis of richness predicted an overall mean of 8243 ± 759 species per sample (range 349–21,092, SD 4804). Good’s coverage ranged from 0.9766 to 0.9934 (mean 0.9837, SD 0.0083). Rarefaction curves for different types of solid waste are presented in Additional file 2. The overall diversity of bacteria populations was high with an average inverse Simpson index of 44.21 ± 1.44, Shannon’s evenness average 4.95 ± 0.02, Chao1 richness estimator of 5926 ± 239, and an abundance based coverage estimator (ACE) of 7202 ± 228.

**Fig. 1:**
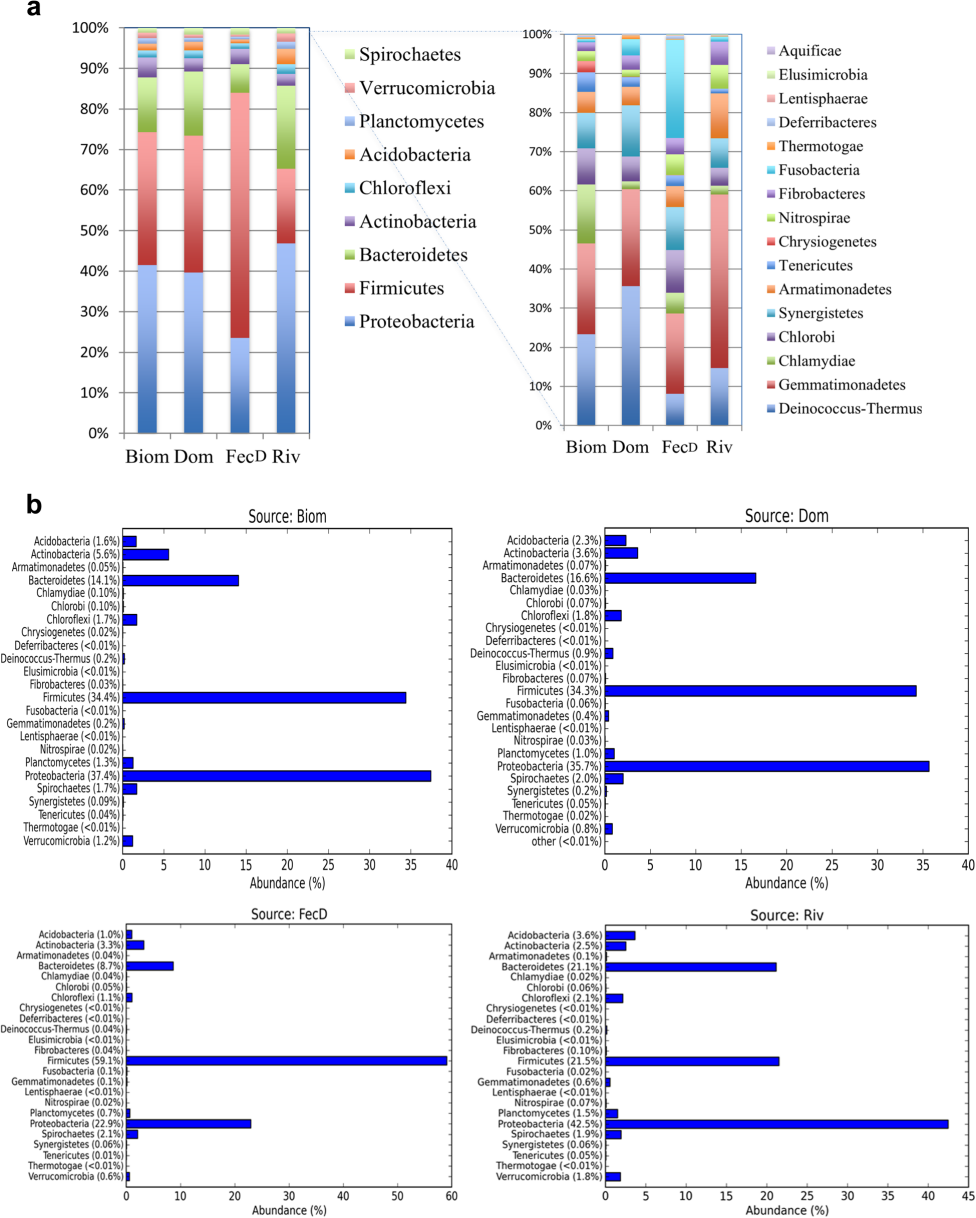
Relative abundance of predominant bacterial phyla on dumpsite. **a** show the overall predominant taxa in different solid wastes pooled together. Different colours represent types of taxa and their relative abundance. **b** show levels of abundance of each predominant taxon when each solid waste was analysed separately. Solid biomedical waste (Biom, *n* =15 domestic solid waste (Dom, *n* = 33), faecal material of pigs on scavenging on dumpsite (FecD, *n* = 20) and in river sludge (Riv, *n* =8). Only phyla in abundance ≥ 0.1 % are shown. Bacterial taxa were assigned at 97 % sequence similarity cut-off level

Thirty-five bacterial phyla were detected, however, only seven of these accounted for more than 93 % of all sequences. The predominant phyla were *Firmicutes* (38 %), *Proteobacteria* (35 %), *Bacteroidetes* (13 %), *Actinobacteria* (3 %), *Acidobacteria* (2 %), *Chloroflexi* (2 %) and *Spirochaetes* (1 %). A large number of phyla were rare and had < 1 % of all sequences (Fig. 1). The unclassified bacteria at phylum level accounted for 2.4 % of all sequences. The heatmap (Additional file 3) shows the abundance and distribution of the predominant bacteria phyla on the dumpsite. When each type of solid waste was separately analysed, *Proteobacteria* was the most predominant in Biom (37.4 %), Dom (35.7 %) and Riv (42.5 %) solid wastes, while *Firmicutes* predominated in FecD (59.1 %) only. Majority of the bacterial phyla in all solid waste were rare and contributed < 0.1 % of all sequences. Biom and FecD had only 11 phyla accounted for 98.2 % and 99.7 % of all sequences, respectively. Likewise in Dom and Riv, 12 phyla accounted for 99.6 % and 99.4 % of all sequences, respectively.

**Fig. 2:**
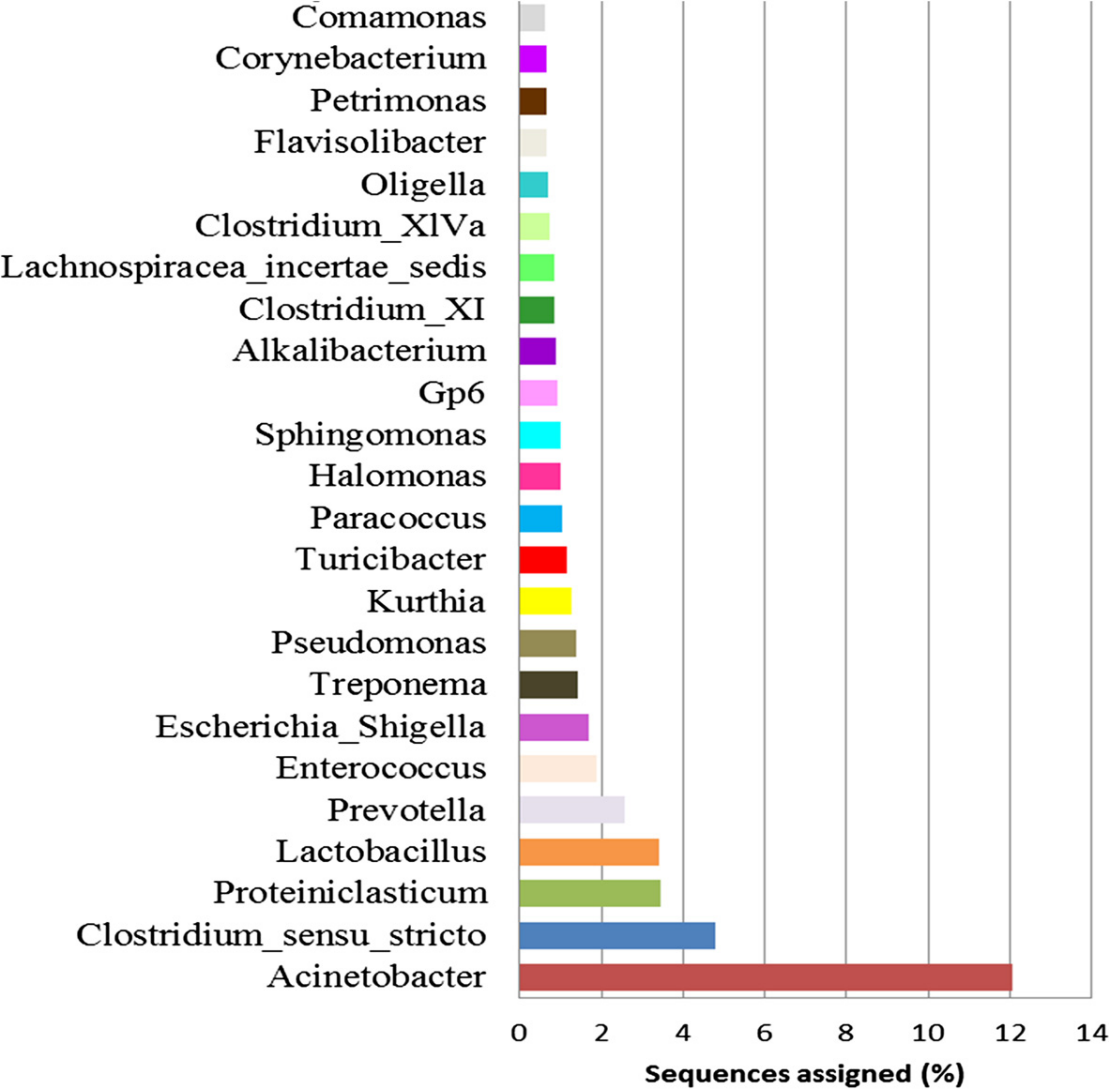
Abundance of predominant bacteria at genus level in the municipal dumpsite. Bar graph depicts the percentage of sequence reads assigned to each taxon at 97 % sequence similarity cut-off

A total of 76,862 OTUs were detected, yet, only 20 % (15,272/76,864) were identified more than ten times. At family level; *Moraxellaceae* (12 %), *Clostridiaceae-1* (8 %), *Ruminococcacea*e (5 %), *Lachnospraceae* (3.7 %) and *Lactobacillaceae* (3.4 %) were the most abundant. Unclassified bacteria accounted for about 14 % of all sequences at family level. A total of 1350 genera were detected, of which 16 % (220/1350) were unclassified. *Acinetobacter* was the most abundant genus accounting for (12.1 %) of all sequences followed by *Clostridium sensu stricto* (4.8 %), *Proteiniclasticum* (3.4 %), *Lactobacillus* (3.4 %), *Prevotella* (2.6 %), *Enterococcus (*2.9 %) and *Escherichia/Shigella* (1.7 %) (Fig. 2).

The core microbiota analyses revealed that none of the OTUs were present across all 78 samples at relative abundance of 1 % or more. Only one OTU, *Acinetobacter* (assigned sequences 688,875) was found in 62 % (48/78) of samples and *Clostridium sensu stricto* (assigned sequences 202,494) in 49 % (38/78) of samples. When each type of solid waste was analysed separately, *Proteiniclasticum* and *Acinetobacter* were found in 67 % (10/15) of Biom; *Clostridium_sensu_stricto* in 80 % (16/20) of FecD; *Acinetobacter* and *Proteiniclasticum* were found in 73 % (24/33) and 55 % (18/33) of Dom, respectively, and in Riv, 80 % (8/10) of samples had *Acinetobacter.* Indicator analysis clearly revealed higher affiliation of some bacterial OTUs to specific solid waste on the dumpsite. Thirteen OTUs were significantly associated with Biom (Indicator value ≥ 70 and *P* ≤ 0.05) amongst which are *Halomonas*, *Alishewanella*, and *Proteiniclasticum*; five were associated to FecD, for example *Enterococcus, Bifidobacterium, Clostridium sensu stricto* and *Cellulosilyticum,* and nine were associated with Riv, such as *Commamonas, Lysobacter* and *Flavobacterium* (Table 1). None of the OTUs were significantly affiliated with Dom at indicator value ≥ 70 and *P* ≤ 0.05.

**Table 1:** Indicator analysis showing bacterial OTUs associated to different solid waste

OTUs	Description (Genus)	#sequences	Indicator value	*P*-value
	Biomedical solid waste (Biom)			
Otu000140	*Idiomarina*	6078	89	0.001
Otu000011	*Halomonas*	83211	86	0.022
Otu000116	*Sporolactobacillaceae_incertae_sedis*	7683	82	0.023
Otu000035	*Tissierella*	18780	80	0.007
Otu000080	*Alkaliflexus*	16213	79	0.004
Otu000136	*Saccharofermentans*	5945	78	0.005
Otu000124	*Alishewanella*	13449	78	0.053
Otu000003	*Proteiniclasticum*	269626	78	0.001
Otu000141	*Unclassified Bacteroidetes*	7998	75	0.001
Otu000411	*Proteiniclasticum*	3303	74	0.006
Otu000525	*Unclassified Bacteroidetes*	346	73	0.001
Otu000567	*Pseudomonas*	2187	71	0.013
Otu000069	*Corynebacterium*	18152	70	0.046
	Faecal material of pigs scavenging on dumpsite (FecD)			
Otu000114	*Clostridium_sensu_stricto*	12444	78	0.039
Otu000477	*Unclassified Lachnospraceae*	2394	78	0.008
Otu000310	*Clostridium_sensu_stricto*	2749	77	0.009
Otu000005	*Enterococcus*	159372	77	0.005
Otu000104	*Bifidobacterium*	11495	73	0.007
	River sludge (Riv)			
Otu000054	*Cloacibacterium*	30207	84	0.051
Otu000807	*Flavobacterium*	2218	84	0.003
Otu000037	*Lysobacter*	35128	81	0.013
Otu001247	*Unclassified Xanthomonadaceae*	849	77	0.001
Otu001672	*Lacibacter*	479	77	0.001
Otu000017	*Commamonas*	54114	77	0.039
Otu001307	*Ferruginibacter*	613	75	0.001
Otu000133	*Dechloromonas*	7673	75	0.039
Otu001398	*Novosphingobium*	571	74	0.001
Otu000596	*Flavobacterium*	2057	73	0.002
Otu001167	*Niabella*	931	72	0.005

None of the OTUs were affiliated to the Dom waste at *p* ≤ 0.05 and indicator value ≥ 70

Of the 35 bacterial phyla detected, Metastats revealed 11 phyla significantly different between Dom and FecD. Further, at genus level, out of 1428 differentially abundant genera, 173 were significantly different between Dom and FecD. When Biom and FecD were compared, 8 phyla and 144 genera were significantly different. The Biom and Dom comparison revealed no difference in bacteria community at phylum level (*P* > 0.05), but 9 bacterial family and 39 genera were significantly different. Of the 1428 genera found in Biom and Dom, 16 % (227/1428) were unclassified. The phylum *Lentisphaerae,* 66 bacterial family and 180 genera were significantly different between Dom and Riv solid waste (Additional file 4).

### Comparison of the bacteria community structure and membership between solid wastes

Phylogenetic tree generated using the Yue & Clayton measures as well as the Jaccard index (Additional file 5) were used in the comparison of the bacterial community structure and membership for different solid wastes. Results of the Parsimony test obtained after the phylogenetic analysis of the Yue and Clayton tree ignoring the branch length revealed a significant difference in bacterial community structure between Dom-FecD (*P* = 0.011) and Dom-Riv (*P* = 0.028). There was no difference in community structure between Biom-Dom (*P* = 0.111), Biom-FecD (*P* = 0.068), Biom-Riv (*P* = 0.5240 and FecD-Riv (*P* = 0.100). When branch length was considered, significantly different structures were found between Dom-Riv (*P* = 0.001) and FecD-Riv (*P* = 0.034), while none was detected between Biom-Dom, Biom-FecD, Dom-FecD and Biom-Riv (*P* > 0.05) using Unweighted UniFrac (Table 2). Further, comparison of the community membership using the phylogenetic tree based on the Jaccard index; the parsimony test revealed that Biom-Dom, Dom-FecD and Biom-Riv (*P* > 0.05) (Table 3) have the same community membership, while in Biom-FecD (*P* = 0.016), Dom-Riv (*P* = 0.002) and FecD-Riv (*P* = 0.002) were different. When Unweighted UniFrac analysis was performed, only Dom-FecD (*P* = 0.039) had significantly different community membership. Analysis of molecular variance revealed a significant similarity in bacterial community between Biom-Dom (Yue & Clayton, *P* = 0.475, Jaccard index, *P* = 0.012), while the rest of the groups were statistically different (*P* < 0.008) (Tables 3 and 4).

**Table 2:** Comparison of bacterial community structure between different solid wastes

	Parsimony		Unweighted UniFrac		Amova	
Groups	ParsScore	ParsSig	UWscore	UWSig	FScore	P* - value
Biom-Dom	10	0.111	0.894454	0.052009	2.28212	0.012
Biom-FecD	8	0.068	0.910399	0.085009	3.14737	<0.001
Dom-FecD	10	0.011	0.853535	0.127009	3.38992	0.002
Biom-Riv	7	0.524	0.934552	0.197003	1.92136	0.016
Dom-Riv	6	0.028	0.959495	0.001009	1.80755	0.028
FecD-Riv	6	0.1	0.967847	0.034006	2.46059	0.004

P* = Correction for multiple comparisons (Bonferroni): Significance *P*- value ≤ 0.0083. Analysis was based on phylogenetic tree generated using Yue & Clayton measure

**Table 3:** Comparison of bacteria community membership between different solid wastes

	Parsimony		Unweighted UniFrac		Amova	
Groups	ParsScore	ParsSig	UWScore	UWSig	FScore	P* - value
Biom-Dom	11	0.297	0.982725	0.149009	0.988888	0.475
Biom-FecD	7	0.016	0.972863	0.235009	2.24675	< 0.001
Dom-FecD	12	0.08	0.981599	0.039009	2.39594	< 0.001
Biom-Riv	5	0.061	0.98467	0.055007	1.37949	< 0.005
Dom-Riv	5	0.002	0.98451	0.060009	1.57885	0.004
FecD-Riv	4	0.003	0.976693	0.159006	2.25111	0.001

P* = Correction for multiple comparisons (Bonferroni): significance *P*- value ≤ 0.0083. Analysis was based on phylogenetic tree generated using Jaccard index

**Table 4:** Summary of good quality sequences and diversity indices of bacteria from different solid waste at species level

^b^Samples	Valid reads	OTUs	ACE^a^	Chao1^a^	Shannon^a^	InvSimpson^a^
Dom	3,466,427	26,243	7338 ± 230	6788 ± 244	5 ± 1.0	26.9 ± 0.7
Biom	1,706,442	18,994	8218 ± 249	6756 ± 257	5.1 ± 0.01	54.2 ± 1.3
Riv	926,648	15,025	10,422 ± 283	8219 ± 287	5.6 ± 0.01	58.5 ± 1.2
FecD	2,369,595	16,697	6183 ± 214	5073 ± 226	4.5 ± 0.01	31.2 ± 0.9

*OTUs* Operational taxonomic units, *ACE* abundance based coverage estimator

^a^Calculations were performed using Mothur package with an OTU definition at > 97 % sequence similarity

^b^Samples: Dom—Domestic solid waste; Biom—Solid biomedical waste; Riv—River sludge; FecD—Faecal material of pigs scavenging on dumpsite

**Fig. 3:**
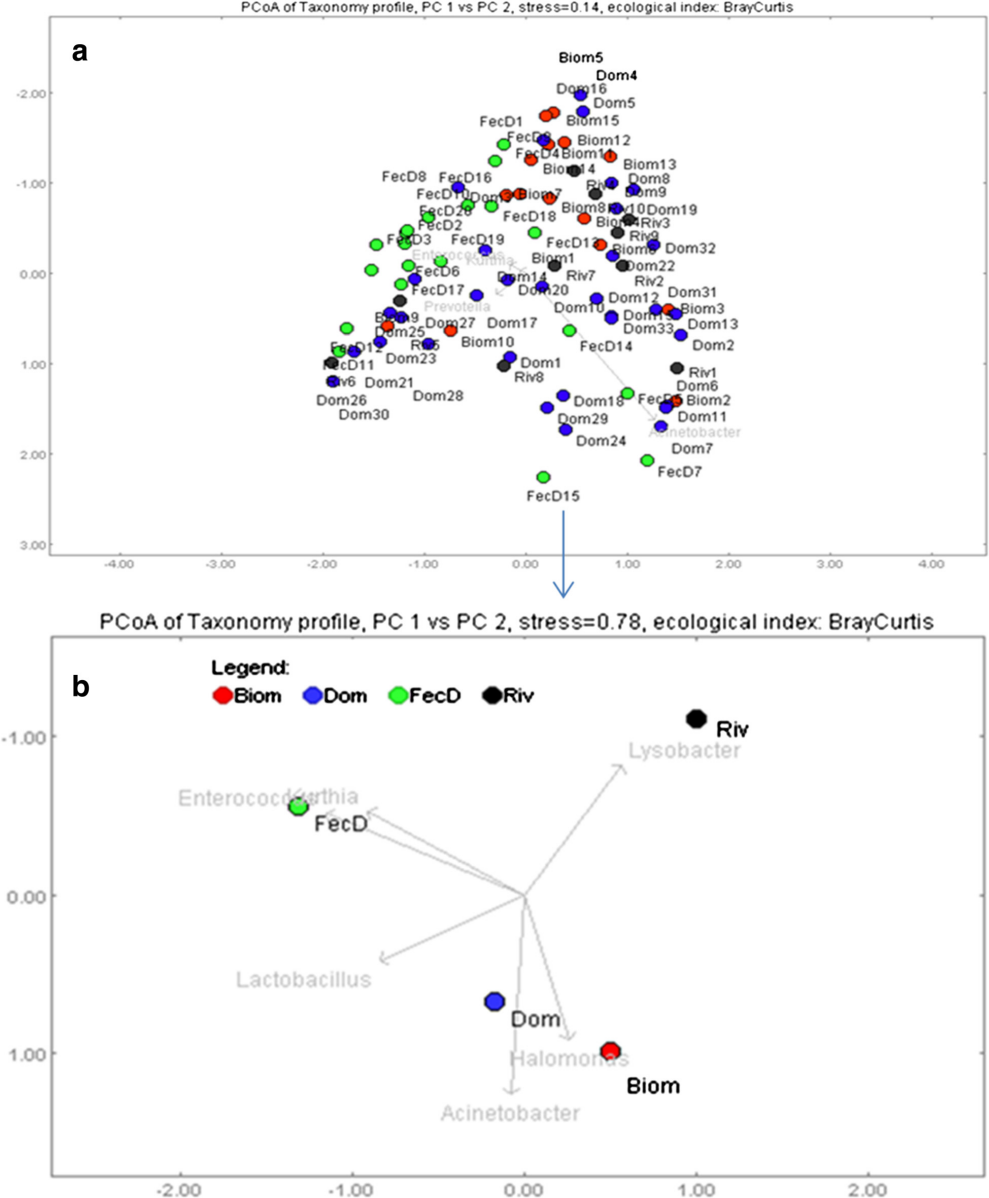
Principal coordinate analysis plots showing clustering of bacterial community from different solid wastes. The PCoA plot was built based on Bray-Curtis dissimilarity distances. **a** was generated from individual samples from different waste types while in (**b**) bacteria population from the same waste type was pooled together. Distances between symbols on the ordination plot reflect relative dissimilarities of bacteria community between solid wastes. The OTUs were estimated at 97 % sequence similarity cut-off

Further, Fig. 3a and b are the graphic representation of the PCoA plot based on Bray-Curtis distances. The spatial separation between centers of the clouds of the bacteria community structure of different solid waste using Amova have shown statistical difference between Biom-FecD (*P* < 0.001), Dom-FecD (*P* = 0.002) and FecD-Riv (*P* = 0.004), but the same community structure between Biom-Dom (*P* = 0.012) which is clearly depicted in a PCoA plot constructed from a pool of bacteria community of the same waste type (Fig. 3b). Bacterial OTUs responsible for the difference in clustering of solid waste types were *Halomonas, Acinetobacter* and *Lactobacillu*s from Biom and Dom solid waste; *Enterococcus* and *Kurthia* in FecD, and *Lysobacte*r in Riv.

## Discussion

This study has identified a substantial abundance and diversity of bacteria in a municipal dumpsite in Arusha, Tanzania. The estimated richness (8243 species) per sample and high diversity (InvSimpson index = 44.21) of bacteria on the dumpsite surpasses many of the previous culture based studies. The detection of 76,862 OTUs from 35 bacterial phyla using high throughput sequencing technique gave a more comprehensive look at the bacterial community on the dumpsite. Of the four predominant phyla, *Firmicutes* was the overall most abundant and includes a variety of gram-positive bacteria. Its particularly dominance in fecal material of pigs is consistent with previous reports by Pajarillo *et al*., [12] as well as in horse fecal microbiota by Costa *et al*., [13] and Shepherd *et al.,* [14]. The predominance of *Firmicutes* in FecD, especially the genus *Clostridium sensu stricto* may be due to the feeding habits of pigs on fibre from a variety of unsorted solid waste as reported by Middelbos *et al*., [15] and Yildirim *et al*.,[16].

The presence of some bacterial genera exclusively to some types of solid waste, justifies a need to sort and treat solid wastes differently. This would deter a possible genetic material exchange, a process which can result in the emergence and re-emergence of new bacteria of public health importance. The revealed affiliation of *Enterococcus* to FecD is consistent with previous findings in fecal material of pigs [17–19]. The potential of *Enterococcus* in nosocomial infections and multidrug resistance [20–22], suggests a need to further examine the antibacterial resistance of isolates from pigs scavenging on dumpsites, and their relation to human and animal pathogens.

The affiliation of *Halomonas* (1 %) to Biom is consistent with findings in gold mines [23] and in contaminated heavy metals wastes [24], as both environments are rich in chemicals. *Halomonas* metabolize cyanide [25] and some are reported to carry plasmid *pZM3H* which confers resistance to different chemicals [24]*,* leading to their application in soil remediation [25, 26]. The fact that these bacteria are predominant, it would be worthwhile to study their roles in the dumpsite, and examining if they contain plasmids that confer resistance to different chemicals. This could lead into their application in the control of chemical pollutants, especially cyanide at the dumpsite.

*Proteobacteria* was the predominant second overall phylum, and the most abundant in Biom, Dom and Riv solid wastes. Several studies have linked the predominance of this phylum with human and animals diseases [13, 27, 28]. At the genus level, the overall predominance of *Acinetobacter* (12 %) on the dumpsite is consistent with report by Saini *et al*., [29] and Hossein *et al*., [30] in solid waste. Previous studies report some species of *Acinetobacter* associated with human and animal’s diseases. A case example is the existence of *Acinetobacter* in clinical isolates from intensive care unit [31], animal with urinary tract infections [32], and as a causative agent of the nosocomial outbreak in Spain [33]. Its predominance in such extreme environment may be linked to their re-counted capacity to detoxify chemicals as well as their multidrug resistance [33–35] which would support their survival in an environment with diverse chemicals from unsorted solid wastes.

*Escherichia* and *Shigella* spp. were among the predominant genera on the dumpsite. These bacteria were also reported in several culture based studies [36–38]. Their capacity to acquire multidrug resistance and their association to human and animal pathogens is well known [39–41]. The abundance of these bacteria within the dumpsite with such extraordinary interaction between animals, humans and microbes may be causing potential health risks. The predominance of these bacteria at the dumpsite underscores a need to study on whether a link exists between bacteria on the dumpsite and the known pathogens. Understanding the potential health risks associated with bacteria from the dumpsite would improve the health of not only the humans interacting with the dumpsite, but with all others that may come in contact with them or other animals.

The significant similarity in bacterial community structure and membership between Biom-Dom, Biom—Riv, and FecD—Riv, implies that similar types of bacteria are found in multiple types of solid wastes. This may be attributed to random disposal of unsorted wastes in the same dumpsite and hence, exchange of bacteria between them. Despite all samples were from the same dumpsite whereby exchange of microbes between solid wastes is possible; distinct affiliation of some bacteria OTUs exclusively to some waste was evident. This phenomenon implies that some bacteria have specific nutritional requirement to survive. It would be interesting to study the bacterial community of sorted solid wastes to examine changes in abundance and diversity.

## Conclusion

We report an ever rich and diverse bacterial community in Arusha municipal dumpsite. The species richness reported here shows the complexity of this man-made ecosystem and calls for further research to assess for a link between human diseases and the dumpsite. Understanding the role of the bacteria within the dumpsite and bacteria found within different types of waste will provide insight into proper disposal of the waste, as well as, limit the risks to human health associated with the dumpsite.

## Methods

### Study site and samples

The site for this study was the Arusha municipal dumpsite, where waste from different urban sources is thrown. Sampling was performed in March through June of 2013, whereby prior to sample collection, a qualitative survey was conducted to identify types of most common waste on the dumpsite as these wastes have influence on microbial composition. This comprised waste from households and markets (foods, pampers, clothes, etc.), chemical and biomedical waste (drug containers, used syringes), various plastics and used glassware, waste from abattoirs and brewers, as well as fecal matter from animals scavenging on the dump itself. Samples for this study were the fresh fecal material of pigs scavenging on the dump (FecD, *n* = 20), domestic solid waste (Dom, *n* = 33), solid biomedical waste (Biom, *n* = 15), and run-off water sludge adjoining a nearby river (Riv, *n* = 8). The core of fresh fecal materials of pigs as well as solid waste and sludge were collected into sterile plastic containers, and, within 1 h, the samples were transported on ice to the laboratory, where total DNA was extracted.

### Extraction of total genomic DNA

Total genomic DNA was extracted from about 250 mg of solid waste samples using PowerSoil™ DNA extraction kit (MOBIO Laboratories, Carlsbad, CA) according to the manufacturer’s protocol. Quality and quantity of total DNA was verified with a NanoDrop ND-2000c spectrophotometer (Thermo Scientific) and gel electrophoresis run in 0.8 % agarose and visualized by ultraviolet illumination after staining with gel red™. The DNA was stored at -20^o^C until further processing.

### 16S rRNA amplification, Library Construction and Sequencing

The Illumina sequencing preparation guide [42] was used to prepare a pooled amplicon of the v4 region of 16S rRNA gene for sequencing. Primers (515 F/806R) designed for v4 region of 16S rRNA and protocols were adapted from Caporaso [43]. Duplicate reactions were done in PCR master mix reaction in 20 μl AccuPower® Taq PCR PreMix composed of 0.5 μl of 10pmol/μl each for the forward and reverse primers, 17 μl molecular grade water, and 2 μl DNA template. The PCR program was run on GeneAMP™ PCR system 9700 set at 95 ^o^C for 3 min, 35 cycles of 94 ^o^C for 45 s, 50 ^o^C for 60 s and 72 ^o^C for 90 s and a final extension at 72 ^o^C for 10 min. Amplicon quality was visualized using gel electrophoresis, and then pooled and purified using QIAquick® PCR purification kit (Qiagen, German) following manufacturer’s protocol. Purified PCR products were normalized to 120 ng. DNA was quantified using Qubit® dsDNA assay kit in Qubit fluorometer 2.0 (Invitrogen, Life Technologies) and the quality was assessed using Agilent DNA 1000 Chip in Agilent 2100 Bioanalyzer (Agilent Technologies, Waldbronn, Germany.

Library denaturing, dilution, and PhiX control preparation was done as described in the 16S metagenomic sequencing library preparation guide [42]. Libraries were denatured and primers were used according to the method described in Caporaso [43]. Sequencing of the library was performed with the Illumina MiSeq platform (San Diego, USA) using 2 × 250 paired- end chemistry at the BecA—ILRI Hub genomic platform, Nairobi, Kenya.

### Sequence data analysis and statistics

The Mothur package algorithms (v1.34.1) were used for both quality control and sequence data analysis [44]. After paired end reads were assembled, sequences were aligned with the Silva 16S rRNA reference database (www.arb-silva.de) [45]. Sequences that were < 239 bp and > 260 bp in length, contained >2 ambiguous base calls or long runs (>8 bp) of homopolymers, or did not align with the correct region were removed. Chimeras were identified using Uchime [46] and eliminated. Taxonomy was assigned using the RDP taxonomy database (http://rdp.cme.msu.edu/index.jsp) [47]. Sequences were binned into operational taxonomic units (OTUs) at 97 % sequence similarity cut-off.

Species richness was assessed with Chao1 [48] and abundance based coverage estimator ACE [49] while evenness and diversity of species were estimated by Shannon [50], jackknife [51] and inverse Simpson [52] indices as well as catchall analysis [53]. All analyses were performed using built-in commands in Mothur v1.34.1. Rarefaction analyses were done at a maximum of 97 % sequence similarity cut-off and was plotted using Phyloseq package [54] in R version 3.1.2. In order to compare bacterial populations between different solid wastes in the same municipal dumpsite, subsampling of sequences from different wastes was done to normalize them for efficient comparison [55]. This consisted of random selection of a number of sequences from each sample consistent to the lowest abundance in all samples. The community membership was compared using the traditional Jaccard index, while population structure was assessed using the Yue & Clayton measure of dissimilarity. Dendrograms were created using Mothur to compare the similarity of bacterial populations among all sample types using both Jaccard index and Yue & Clayton measure which account for the relative abundances in each sample. Figures were generated by FigTree v1.4.2. [56].

To check if the bacterial communities differed significantly between solid wastes, the parsimony [57] and Unweighted UniFrac [58] tests were done. The MOTHUR commands “parsimony” and “Unifrac.Unweighted”, respectively, were applied to the Jaccard and the Yue & Clayton OTU based phylogenetic tree. The statistical significance of the difference in genetic diversity of bacteria community within each solid waste type from the average genetic diversity of both communities pooled together was also assessed using Analysis of Molecular Variance (AMOVA) [59].

The core microbiota analysis was performed in Mothur with command “get.coremicrobiome” and it consisted of identification of OTUs (at relative abundance of 1 %) present in all samples when pooled together and also when each type of solid waste was individually analysed. Indicator analysis [60] was used to test for possible OTUs affiliated to different types of solid waste. Indicator values (IV) ranged from 1 to 100 with higher values for stronger indicators. Though literature considers indicator values > 30 and *P*-value ≤ 0.05 as good indicators [60], in this study only OTUs with indicator values ≥ 70 and *P* ≤ 0.05 were judged as having strong affiliation to particular solid waste. The Metastats program [61] was used to identify statistically different OTUs among solid wastes. The shared OTUs file, consensus taxonomy file, and metadata file generated in Mothur v.1.34.1 were imported into METAGENassist [62] where visualization using a heatmap, bar charts and PCA plots was done get more insights on the nature of bacteria present on dumpsite. The BIOM file generated in Mothur was imported into MEGAN5 v5.5.3 [63] where Principal coordinate analysis and relative abundance of different taxa were visualized. A *p*-value of ≤ 0.05 was considered significant for all comparisons.

## Abbreviations

ABCF, African Biosciences Challenge Fund; BecA—ILRI, Biosciences eastern and central Africa—International Livestock Research Institute; MEGAN, MetaGenome ANalyser; OTUs, operational taxonomic units

## Additional files

Additional file 1: Table S1. Showing different animals and solid wastes on the dumpsite. (DOCX 13 kb) Additional file 2: Rarefaction analysis curves of different solid waste from the dumpsite. (DOCX 129 kb) Additional file 3: Heatmap showing abundance and distribution of predominant bacteria phyla from different solid waste samples. (DOCX 125 kb) Additional file 4: Tables showing significantly different bacteria at genus level between different types of wastes solid. (DOCX 55 kb) Additional file 5: OTU based dendrogram demonstrating the similarity and differences of bacteria communities’ structure and membership from different solid wastes. Results were obtained using the Yue & Clayton measure (Fig a) and the Jaccard index (Fig b). (DOCX 1469 kb)
