# Supplementary material for: High-throughput sequencing of 16S rRNA Gene Reveals Substantial Bacterial Diversity on the Municipal Dumpsite
Source: BMC Microbiol. 2016 Jul 11;16:145. doi: 10.1186/s12866-016-0758-8 (PMC4940873; doi:10.1186/s12866-016-0758-8)
Supplement: Additional file 1: Table S1. — Showing different animals and solid wastes on the dumpsite. (DOCX 13 kb) [file 12866_2016_758_MOESM1_ESM.docx]

Table S1: Types of organisms and solid waste found in Arusha municipal dumpsite, Tanzania

| Organisms interacting on the dumpsite | | Variety of solid waste on the dumpsite | | |
| --- | --- | --- | --- | --- |
| Description | Estimate | Biomedical | Domestic | Industrial |
| Human  Animals  Pigs  Cattle  Goat  Donkey  Dogs  Sheep  Rodents  Insects  Mosquitoes  Cockroches  Flies  Birds  Crows  Storks  Chicken  Ducks | 20 – 30  150 – 200  10 -15  5 -10  2 -4  5 -10  3 -7  5 -10  Many  Many  Many  10 -20  7 -14  20 -30  2- 4 | Used syringes  Used swabs  Used needles  Expired grugs  Drug containers  Catheters  Oxygen masks | Diapers  Food remains  Worn clothes  Fruits and vegs peels  Worn utensils  Expired cosmetics  Dead animals | Plastic and glass bottles  Tetrapacks  Cardboard boxes  Broken bottles  Brewery filter mash  Abbatoirs waste (hides and skins) |

The table show the average population size of animals, insects and birds, as well as variety of solid waste as over a period of two weeks of qualitative survey of the Arusha municipal dumpsite
