# Supplementary material for: High-throughput sequencing of 16S rRNA Gene Reveals Substantial Bacterial Diversity on the Municipal Dumpsite
Source: BMC Microbiol. 2016 Jul 11;16:145. doi: 10.1186/s12866-016-0758-8 (PMC4940873; doi:10.1186/s12866-016-0758-8)
Supplement: Additional file 2: — Rarefaction analysis curves of different solid waste from the dumpsite. (DOCX 129 kb) [file 12866_2016_758_MOESM2_ESM.docx]

Rarefaction curves of different solid waste on the municipal dumpsite

| 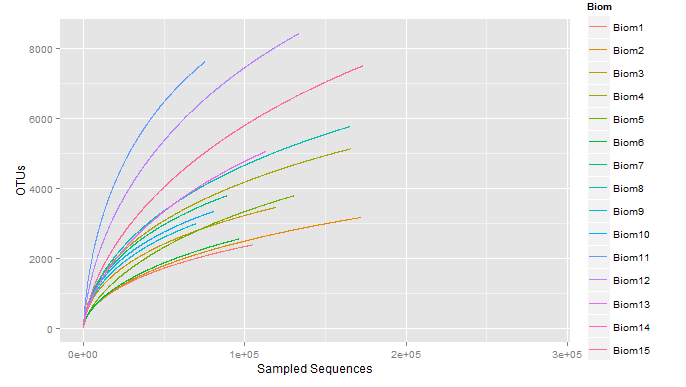  (a) | 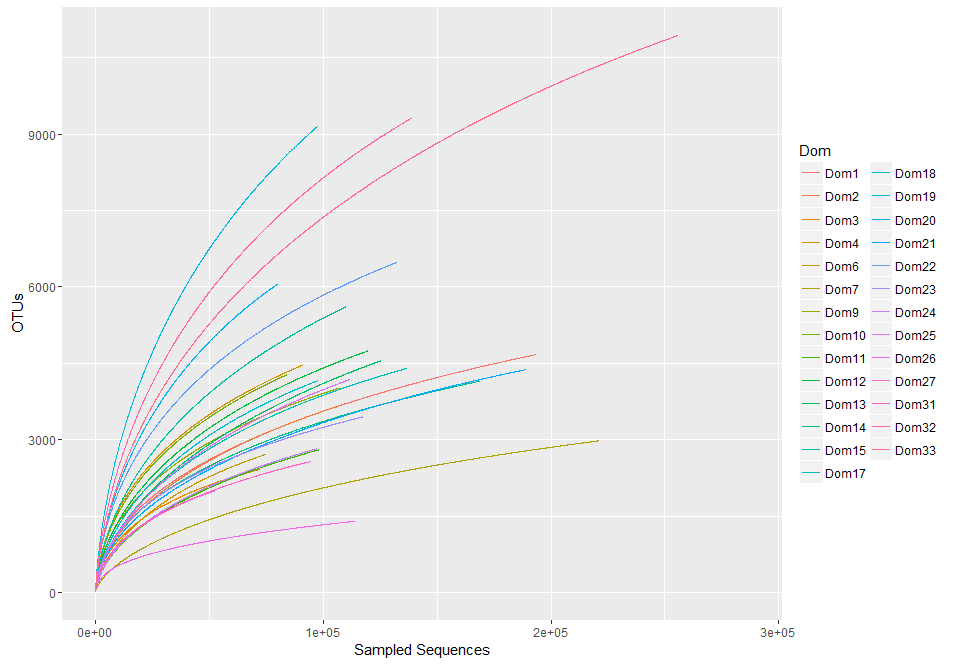  (b) |
| --- | --- |
| 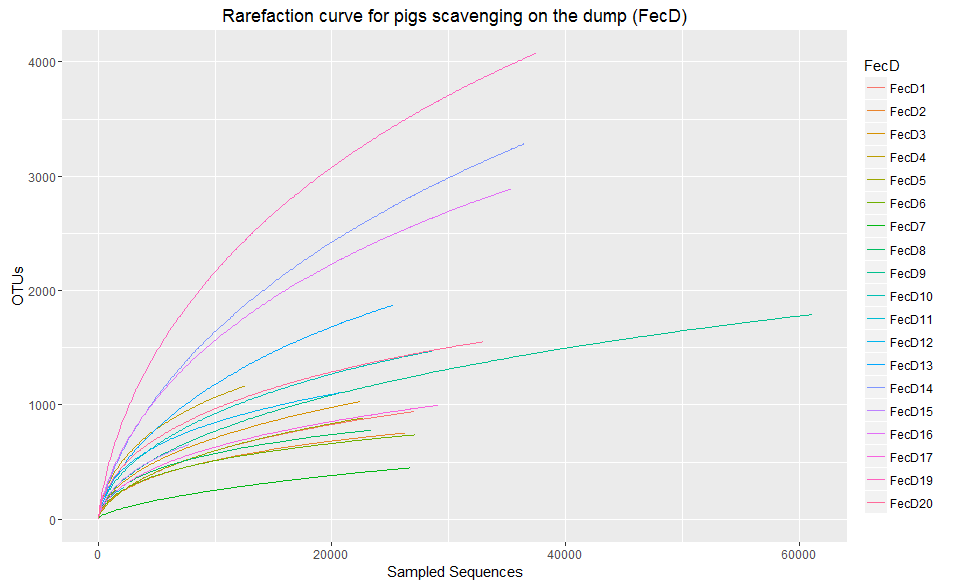  (c) | 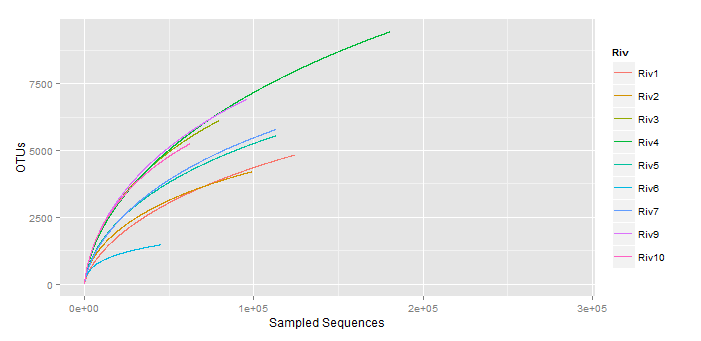  (d) |

Figure S1: Rarefaction analysis of the different solid waste. Estimation of OTUs was done at 97% sequence similarity cut-off. (a) Represent solid biomedical waste, (b) domestic waste (c) faecal material of pigs scavenging on dumpsite and (d) river sludge near the municipal dumpsite. No subsampling was done to any type of solid waste.
