# Supplementary material for: High-throughput sequencing of 16S rRNA Gene Reveals Substantial Bacterial Diversity on the Municipal Dumpsite
Source: BMC Microbiol. 2016 Jul 11;16:145. doi: 10.1186/s12866-016-0758-8 (PMC4940873; doi:10.1186/s12866-016-0758-8)
Supplement: Additional file 3: — Heatmap showing abundance and distribution of predominant bacteria phyla from different solid waste samples. (DOCX 125 kb) [file 12866_2016_758_MOESM3_ESM.docx]

Heatmap showing abundance and distribution of predominant bacteria phyla from different solid waste


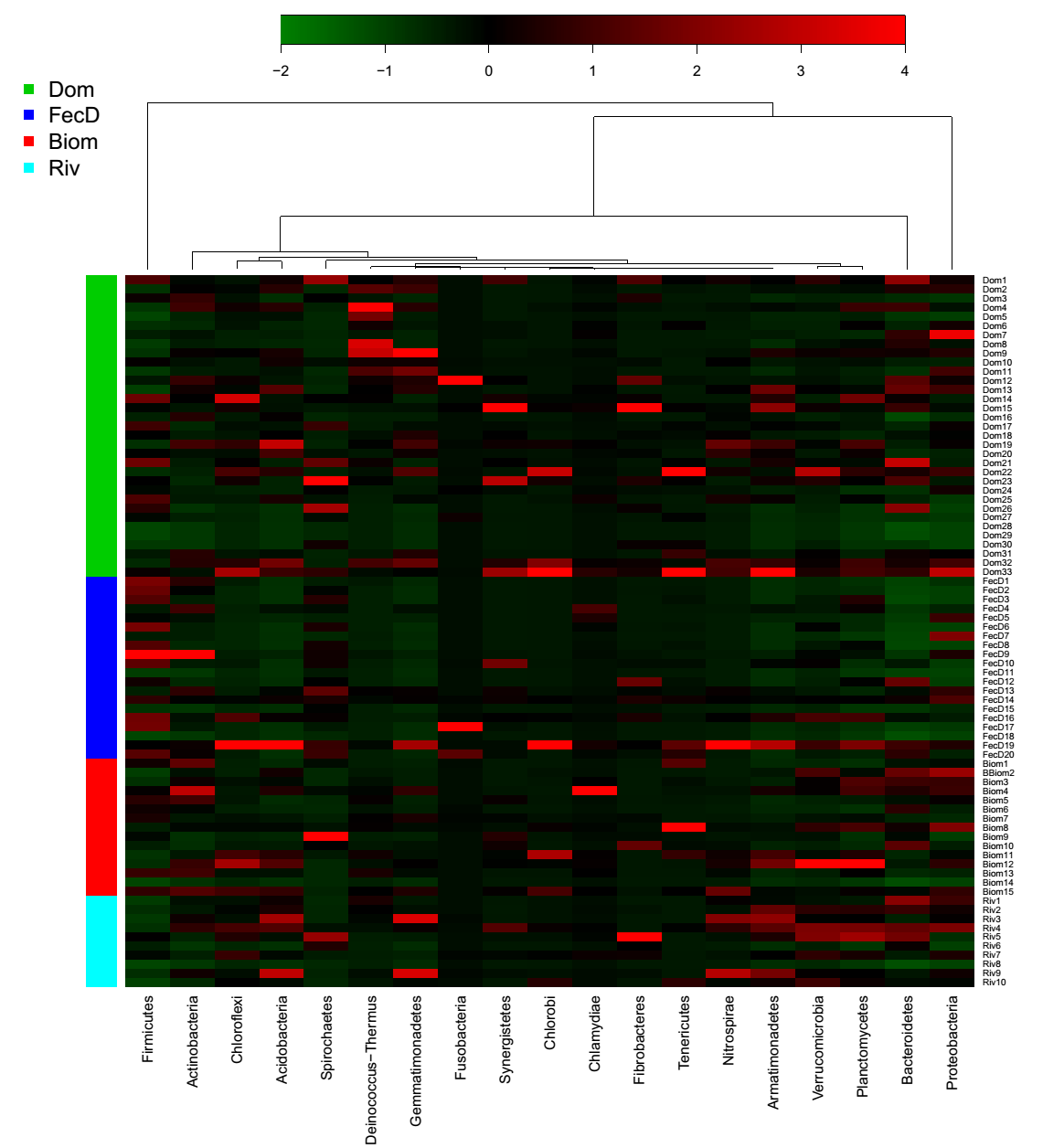


Figure S1: Heatmap showing abundance and distribution of predominant bacteria phyla from different types of solid waste. Taxa were assigned at 97% 16S rRNA sequence similarity. The abundance of each phylum is indicated by a different colour as shown in key.
