# Supplementary material for: High-throughput sequencing of 16S rRNA Gene Reveals Substantial Bacterial Diversity on the Municipal Dumpsite
Source: BMC Microbiol. 2016 Jul 11;16:145. doi: 10.1186/s12866-016-0758-8 (PMC4940873; doi:10.1186/s12866-016-0758-8)
Supplement: Additional file 4: — Tables showing significantly different bacteria at genus level between different types of wastes solid. (DOCX 55 kb) [file 12866_2016_758_MOESM4_ESM.docx]

Table S1: Significantly different bacteria at genus level between Biom and Dom solid waste

| S/N | Genera | Mean_ Biom | mean Dom | P-value |
| --- | --- | --- | --- | --- |
| 1 | *5_genus_incertae_sedis* | 0.002426508 | 0.003941834 | 0.046 |
| 2 | *Afipia* | 6.52E-05 | 0 | 0.004 |
| 3 | *Aidingimonas* | 3.97E-05 | 0 | 0.036 |
| 4 | *Akkermansia* | 0.00015699 | 0.000409762 | 0.03 |
| 5 | *Alkalimonas* | 4.84E-05 | 0 | 0.036 |
| 6 | *Allofustis* | 6.53E-05 | 0 | 0.001 |
| 7 | *Anaeroplasma* | 4.43E-05 | 0.000299431 | 0.004 |
| 8 | *Anaerotruncus* | 0.000106643 | 0.000296284 | 0.019 |
| 9 | *Anoxynatronum* | 7.04E-05 | 3.26E-06 | 0.044 |
| 10 | *Atopobium* | 6.54E-05 | 7.20E-06 | 0.044 |
| 11 | *Duganella* | 0.000149495 | 2.94E-05 | 0.02 |
| 12 | *Gordonibacter* | 0.000159673 | 3.78E-05 | 0.05 |
| 13 | *Haematobacter* | 7.53E-05 | 0 | 0.012 |
| 14 | *Haliangium* | 0.001044774 | 0.000721728 | 0.035 |
| 15 | *Haliscomenobacter* | 0.000258662 | 0.000457528 | 0.014 |
| 16 | *Halothiobacillus* | 0.000173944 | 4.41E-05 | 0.002 |
| 17 | *Janthinobacterium* | 0 | 7.30E-05 | 0.02 |
| 18 | *Lewinella* | 0.000465988 | 0.000825868 | 0.03 |
| 19 | *Massilia* | 7.95E-06 | 7.07E-05 | 0.045 |
| 20 | *Microcella* | 5.57E-05 | 8.22E-06 | 0.017 |
| 21 | *Millisia* | 0.000109893 | 2.18E-05 | 0.007 |
| 22 | *Oligella* | 0.000325157 | 0.000694592 | 0.045 |
| 23 | *Peptoniphilus* | 0.000104073 | 0 | 0.004 |
| 24 | *Perlucidibaca* | 0.000183572 | 5.72E-05 | 0.015 |
| 25 | *Propionivibrio* | 8.55E-05 | 9.66E-06 | 0.001 |
| 26 | *Pseudorhodoferax* | 0.000150644 | 0 | 0 |
| 27 | *Schlesneria* | 9.14E-05 | 3.26E-06 | 0.001 |
| 28 | *Solimonas* | 0.00015094 | 5.24E-05 | 0.028 |
| 29 | *Sphingobium* | 0.001195233 | 0.000629521 | 0.048 |
| 30 | *Sporobacter* | 0.003330822 | 0.005496117 | 0.048 |
| 31 | *Sporosalibacterium* | 4.40E-05 | 0 | 0.036 |
| 32 | *Sulfuritalea* | 3.25E-05 | 0 | 0.036 |
| 33 | *Waddlia* | 0.000135668 | 4.76E-05 | 0.021 |
| 34 | *Xiphinematobacter* | 0.000177622 | 6.94E-05 | 0.015 |

Table S2: Significantly different bacteria at genus level between Biom and FecD wastes

| S/N | feature | mean-Biom | Mean_FecD | p-val |
| --- | --- | --- | --- | --- |
| 1 | *5_genus_incertae_sedis* | 0.002426508 | 0.005089808 | 0.001 |
| 2 | *Acetanaerobacterium* | 0.000583545 | 0.001421545 | 0.013 |
| 3 | *Acetitomaculum* | 5.52E-05 | 0.000391802 | 0.001 |
| 4 | *Acetobacterium* | 0.000290781 | 0.000123016 | 0.049 |
| 5 | *Achromobacter* | 8.13E-05 | 0 | 0.031 |
| 6 | *Acidaminobacter* | 8.84E-05 | 0.000199251 | 0.04 |
| 7 | *Aciditerrimonas* | 0.002123494 | 0.001172092 | 0.027 |
| 8 | *Aerococcus* | 0.00031643 | 0.000799141 | 0.011 |
| 9 | *Aggregatibacter* | 2.63E-05 | 0.000161169 | 0.035 |
| 10 | *Akkermansia* | 0.00015699 | 0.000739684 | 0.002 |
| 11 | *Algoriphagus* | 0.000799868 | 0.000368239 | 0.03 |
| 12 | *Altererythrobacter* | 0.001082274 | 0.000605289 | 0.039 |
| 13 | *Alterococcus* | 0.000182825 | 4.21E-05 | 0.009 |
| 14 | *Anaerobranca* | 7.94E-05 | 0 | 0.031 |
| 15 | *Anaeromyxobacter* | 0.001439154 | 0.000599278 | 0.004 |
| 16 | *Anaeroplasma* | 4.43E-05 | 0.000384874 | 0.001 |
| 17 | *Anaerostipes* | 0.00019243 | 0.000793852 | 0.009 |
| 18 | *Anaerovibrio* | 0.000205033 | 0.000634306 | 0.01 |
| 19 | *Aquiflexum* | 0.000309648 | 0.000140051 | 0.036 |
| 20 | *Azospirillum* | 0.000503889 | 0.000188962 | 0.023 |
| 21 | *Azotobacter* | 0.000324228 | 0.000135911 | 0.032 |
| 22 | *Bacteriovorax* | 0.000407831 | 0.000133989 | 0.044 |
| 23 | *Bacteroides* | 0.002604564 | 0.005023329 | 0.019 |
| 24 | *Barnesiella* | 0.001193624 | 0.003575533 | 0.001 |
| 25 | *Bdellovibrio* | 0.001165384 | 0.000378753 | 0.01 |
| 26 | *Bifidobacterium* | 0.000606521 | 0.001655834 | 0.006 |
| 27 | *Blastopirellula* | 0.006694716 | 0.002605901 | 0.009 |
| 28 | *Blautia* | 0.002548571 | 0.006993896 | 0.001 |
| 29 | *Brachymonas* | 0.000259213 | 0.000106128 | 0.016 |
| 30 | *BRC1_genera_incertae_sedis* | 0.001521976 | 0.000607712 | 0.016 |
| 31 | *Brevibacillus* | 0.00039301 | 0.000112637 | 0.025 |
| 32 | *Bulleidia* | 0.000447597 | 0.001575639 | 0.001 |
| 33 | *Butyrivibrio* | 6.89E-05 | 0.000242104 | 0.017 |
| 34 | *Caldilinea* | 0.002985043 | 0.001367198 | 0.017 |
| 35 | *Campylobacter* | 0.000194295 | 0.000478345 | 0.005 |
| 36 | *Caryophanon* | 0 | 0.000156577 | 0.028 |
| 37 | *Cellulomonas* | 0.000282331 | 0.000621455 | 0.041 |
| 38 | *Cellulosilyticum* | 0.001234297 | 0.003434429 | 0.003 |
| 39 | *Chlamydia* | 3.53E-05 | 0.000280499 | 0.011 |
| 40 | *Chondromyces* | 0.001410409 | 0.000693577 | 0.015 |
| 41 | *Clostridium_IV* | 0.003907005 | 0.010845164 | 0.002 |
| 42 | *Clostridium_sensu_stricto* | 0.008737839 | 0.019360076 | 0.008 |
| 43 | *Clostridium_XI* | 0.001556673 | 0.004209043 | 0.001 |
| 44 | *Clostridium_XlVa* | 0.00776595 | 0.015202681 | 0.003 |
| 45 | *Clostridium_XlVb* | 0.000940319 | 0.00179939 | 0.044 |
| 46 | *Conexibacter* | 0.002506939 | 0.001257355 | 0.007 |
| 47 | *Coprobacillus* | 4.07E-05 | 0.00027902 | 0.007 |
| 48 | *Coprococcus* | 0.001146161 | 0.002609547 | 0.006 |
| 49 | *Coxiella* | 0.000385615 | 0.000131023 | 0.041 |
| 50 | *Dasania* | 0.000114035 | 1.04E-05 | 0.039 |
| 51 | *Desulfocapsa* | 0.000280995 | 9.77E-05 | 0.019 |
| 52 | *Desulfomicrobium* | 0.00037522 | 0.000142273 | 0.01 |
| 53 | *Desulfonatronum* | 9.61E-05 | 0 | 0.016 |
| 54 | *Desulfonispora* | 1.76E-05 | 8.22E-05 | 0.035 |
| 55 | *Dialister* | 8.59E-05 | 0.000204914 | 0.045 |
| 56 | *Dorea* | 0.001238467 | 0.002564325 | 0.004 |
| 57 | *Dysgonomonas* | 0.00273743 | 0.000530496 | 0.015 |
| 58 | *Elusimicrobium* | 8.84E-05 | 0.000362769 | 0.032 |
| 59 | *Ensifer* | 0.000992385 | 0.000361396 | 0.038 |
| 60 | *Enterorhabdus* | 0.000138352 | 0.000341413 | 0.04 |
| 61 | *Erysipelotrichaceae_incertae_sedis* | 0.000450977 | 0.001462786 | 0.001 |
| 62 | *Ethanoligenens* | 0.000398183 | 0.000896362 | 0.012 |
| 63 | *Eubacterium* | 0.001034209 | 0.001716617 | 0.045 |
| 64 | *Euzebya* | 0.000595835 | 0.000183618 | 0.011 |
| 65 | *Faecalibacterium* | 0.001034857 | 0.002859885 | 0.004 |
| 66 | *Ferruginibacter* | 0.00100485 | 0.000243701 | 0.03 |
| 67 | *Filomicrobium* | 0.00023388 | 7.81E-05 | 0.029 |
| 68 | *Flavihumibacter* | 0.000841979 | 0.000289566 | 0.022 |
| 69 | *Flavonifractor* | 0.000460392 | 0.001273833 | 0.005 |
| 70 | *Geminicoccus* | 0.002718103 | 0.001407646 | 0.01 |
| 71 | *Gp4* | 0.007847593 | 0.00476597 | 0.036 |
| 72 | *Haliangium* | 0.001044774 | 0.000350226 | 0.001 |
| 73 | *Haliea* | 0.000559794 | 0.000254208 | 0.03 |
| 74 | *Haloferula* | 0.000472279 | 8.21E-05 | 0.001 |
| 75 | *Helicobacter* | 0.000171411 | 0.000440455 | 0.022 |
| 76 | *Heliothrix* | 0.00078312 | 0.000179573 | 0.007 |
| 77 | *Hespellia* | 0.000183968 | 0.000340507 | 0.033 |
| 78 | *Holophaga* | 1.59E-05 | 7.06E-05 | 0.02 |
| 79 | *Howardella* | 5.02E-05 | 0.000428798 | 0 |
| 80 | *Ilumatobacter* | 0.000971178 | 0.000444624 | 0.017 |
| 81 | *Indibacter* | 0.000307953 | 7.43E-05 | 0.025 |
| 82 | *Inquilinus* | 0.000217073 | 6.29E-05 | 0.01 |
| 83 | *Isobaculum* | 1.32E-05 | 0.000159372 | 0.035 |
| 84 | *Klebsiella* | 0 | 7.66E-05 | 0.014 |
| 85 | *Kofleria* | 0.000383076 | 0.00010108 | 0.004 |
| 86 | *Lachnospiracea_incertae_sedis* | 0.002968346 | 0.008519407 | 0.001 |
| 87 | *Lactobacillus* | 0.003576022 | 0.006459193 | 0.02 |
| 88 | *Levilinea* | 0.000512346 | 0.000153702 | 0.012 |
| 89 | *Mitsuokella* | 0.000232957 | 0.000702384 | 0.012 |
| 90 | *Mogibacterium* | 0.000718045 | 0.001508814 | 0.04 |
| 91 | *Nannocystis* | 0.000375784 | 0.000204572 | 0.013 |
| 92 | *Natronincola* | 0.00018337 | 2.08E-05 | 0.013 |
| 93 | *Nitriliruptor* | 0.001240434 | 0.000450545 | 0.008 |
| 94 | *Nitrosomonas* | 0.00051806 | 7.05E-05 | 0.003 |
| 95 | *Nitrospira* | 0.000939735 | 0.000416037 | 0.016 |
| 96 | *Oligella* | 0.000325157 | 0.000847594 | 0.004 |
| 97 | *Ornithinibacillus* | 8.37E-05 | 0 | 0.031 |
| 98 | *Oscillibacter* | 0.002316169 | 0.006724864 | 0.002 |
| 99 | *Papillibacter* | 0.001451009 | 0.002683703 | 0.032 |
| 100 | *Paraeggerthella* | 0.000127749 | 0.000446858 | 0.028 |
| 101 | *Parapedobacter* | 0.001050208 | 0.00015628 | 0.006 |
| 102 | *Paraprevotella* | 0.000629754 | 0.001798605 | 0.013 |
| 103 | *Pasteuria* | 0.004959617 | 0.002365603 | 0.041 |
| 104 | *Pediococcus* | 0.000171146 | 0.000484569 | 0.021 |
| 105 | *Peptococcus* | 0.000443701 | 0.000865515 | 0.035 |
| 106 | *Planctomyces* | 0.008608864 | 0.003534475 | 0.002 |
| 107 | *Plesiocystis* | 0.000365227 | 6.44E-05 | 0.002 |
| 108 | *Porphyrobacter* | 0.001104133 | 0.000459605 | 0.041 |
| 109 | *Porticoccus* | 0.000321678 | 8.08E-05 | 0.007 |
| 110 | *Prevotella* | 0.007005839 | 0.01596701 | 0.007 |
| 111 | *Proteiniclasticum* | 0.010281141 | 0.001920867 | 0.009 |
| 112 | *Proteiniphilum* | 0.000800115 | 0.000223395 | 0.002 |
| 113 | *Proteocatella* | 0.000350909 | 7.47E-05 | 0.03 |
| 114 | *Pseudobutyrivibrio* | 0.000231659 | 0.000675493 | 0.011 |
| 115 | *Pseudoflavonifractor* | 0.000419625 | 0.000875676 | 0.012 |
| 116 | *Pseudofulvimonas* | 0.000658656 | 0.000331255 | 0.024 |
| 117 | *Pseudoramibacter* | 0.00016755 | 0.00052823 | 0.008 |
| 118 | *Pseudorhodoferax* | 0.000150644 | 1.13E-05 | 0.003 |
| 119 | *Pusillimonas* | 0.001502818 | 0.000612179 | 0.008 |
| 120 | *Pyramidobacter* | 1.98E-05 | 0.000163291 | 0.019 |
| 121 | *Rhodopirellula* | 0.002079498 | 0.001094807 | 0.015 |
| 122 | *Robinsoniella* | 0.000150932 | 0.000509029 | 0.001 |
| 123 | *Roseburia* | 0.000778761 | 0.0025137 | 0.005 |
| 124 | *Ruminococcus* | 0.003709739 | 0.009873302 | 0.001 |
| 125 | *Salegentibacter* | 0.00011536 | 0.000204082 | 0.012 |
| 126 | *Salinibacter* | 0.000752282 | 0.000318732 | 0.012 |
| 127 | *Salisaeta* | 0.001472241 | 0.000595253 | 0.001 |
| 128 | *Sandarakinorhabdus* | 0.000281942 | 0.00013003 | 0.042 |
| 129 | *Sarcina* | 5.77E-05 | 0.000441 | 0.006 |
| 130 | *Selenomonas* | 3.13E-05 | 0.000143218 | 0.035 |
| 131 | *Sharpea* | 0.000119644 | 0.000363381 | 0.033 |
| 132 | *Sinomonas* | 1.76E-05 | 0.00031118 | 0.005 |
| 133 | *Slackia* | 7.31E-05 | 0.000382641 | 0.001 |
| 134 | *Solitalea* | 0.000172218 | 5.15E-05 | 0.035 |
| 135 | *Spartobacteria_genera_incertae_sedis* | 0.005345601 | 0.002713114 | 0.004 |
| 136 | *Sphaerotilus* | 4.20E-05 | 0.000122209 | 0.035 |
| 137 | *Sporobacter* | 0.003330822 | 0.009297008 | 0.001 |
| 138 | *Subdoligranulum* | 0.000328919 | 0.000915867 | 0.01 |
| 139 | *Thiohalophilus* | 0.000235943 | 3.41E-05 | 0.001 |
| 140 | *Treponema* | 0.004574724 | 0.01002677 | 0.001 |
| 141 | *Truepera* | 0.002573534 | 0.001312322 | 0.003 |
| 142 | *Turicibacter* | 0.000944458 | 0.002883276 | 0.003 |
| 143 | *Vampirovibrio* | 0.002162484 | 0.004958706 | 0.003 |
| 144 | *Waddlia* | 0.000135668 | 1.23E-05 | 0.023 |

Table S3: Significantly different bacteria at genus level between Biom and Riv solid wastes

| S/N | Bacterial genera | Mean_Biom | Mean_Riv | P_val |
| --- | --- | --- | --- | --- |
| 1 | *Acetoanaerobium* | 0.00061967 | 0.000222318 | 0.022 |
| 2 | *Acetobacterium* | 0.000290781 | 8.86E-05 | 0.02 |
| 3 | *Acholeplasma* | 0.001430354 | 0.000320976 | 0.006 |
| 4 | *Achromobacter* | 8.13E-05 | 0 | 0.04 |
| 5 | *Alicyclobacillus* | 0.000102063 | 0 | 0.012 |
| 6 | *Alishewanella* | 0.000338792 | 0.000116253 | 0.003 |
| 7 | *Alkaliphilus* | 0.000489367 | 0.000146316 | 0.006 |
| 8 | *Allofustis* | 6.53E-05 | 0 | 0.04 |
| 9 | *Aminomonas* | 0.000142857 | 2.07E-05 | 0.017 |
| 10 | *Ancylobacter* | 8.86E-05 | 0 | 0.04 |
| 11 | *Aquimonas* | 0.000339816 | 0.000556013 | 0.05 |
| 12 | *Armatimonadetes_gp5* | 0.001028273 | 0.002514725 | 0.016 |
| 13 | *Azotobacter* | 0.000324228 | 0.000161228 | 0.025 |
| 14 | *Bacillus* | 0.004259462 | 0.002546007 | 0.02 |
| 15 | *Balneola* | 0.000579132 | 0.000138129 | 0.05 |
| 16 | *Bellilinea* | 0.003279469 | 0.006183781 | 0.033 |
| 17 | *Bordetella* | 0.00025439 | 0.000150645 | 0.026 |
| 18 | *Brevibacillus* | 0.00039301 | 6.22E-05 | 0.003 |
| 19 | *Brevibacterium* | 0.000476087 | 0.000157321 | 0.001 |
| 20 | *Brevundimonas* | 0.001913801 | 0.000870046 | 0.02 |
| 21 | *Caenispirillum* | 0.000168443 | 0 | 0.003 |
| 22 | *Caldalkalibacillus* | 7.22E-05 | 0 | 0.022 |
| 23 | *Castellaniella* | 0.000574975 | 0.000141446 | 0.003 |
| 24 | *Cerasicoccus* | 0.000599666 | 0.00013752 | 0.012 |
| 25 | *Clostridium_XII* | 0.000225394 | 1.80E-05 | 0.029 |
| 26 | *Cohnella* | 0.000138181 | 1.06E-05 | 0.029 |
| 27 | *Corynebacterium* | 0.003438129 | 0.000735171 | 0.003 |
| 28 | *Defluviicoccus* | 9.31E-05 | 0.000335578 | 0.025 |
| 29 | *Demequina* | 0.000575873 | 0.00034179 | 0.033 |
| 30 | *Desmospora* | 0.000380469 | 9.64E-05 | 0.003 |
| 31 | *Desulfitibacter* | 0.000127262 | 1.89E-05 | 0.029 |
| 32 | *Desulfitispora* | 9.27E-05 | 0 | 0.04 |
| 33 | *Desulfitobacterium* | 0.000254379 | 7.70E-05 | 0.031 |
| 34 | *Dethiosulfatibacter* | 0.000120091 | 0 | 0.007 |
| 35 | *Devosia* | 0.002834287 | 0.001332308 | 0.003 |
| 36 | *Dietzia* | 0.000615376 | 0.000306872 | 0.031 |
| 37 | *Dysgonomonas* | 0.00273743 | 0.000561384 | 0.04 |
| 38 | *Ectothiorhodospira* | 0.000134833 | 1.06E-05 | 0.017 |
| 39 | *Ensifer* | 0.000992385 | 0.000341668 | 0.002 |
| 40 | *Erysipelothrix* | 0.000721174 | 4.31E-05 | 0.002 |
| 41 | *Euzebya* | 0.000595835 | 0.000222228 | 0.048 |
| 42 | *Exiguobacterium* | 0.000844862 | 0.000280124 | 0.002 |
| 43 | *Fervidicella* | 8.32E-05 | 0.000370454 | 0.034 |
| 44 | *Fodinicurvata* | 0.000278575 | 0.000107854 | 0.045 |
| 45 | *Garciella* | 0.000411299 | 4.23E-05 | 0.019 |
| 46 | *Geobacillus* | 0.000600794 | 9.65E-05 | 0.001 |
| 47 | *Gp17* | 0.001109287 | 0.002473336 | 0.037 |
| 48 | *Halothiobacillus* | 0.000173944 | 1.63E-05 | 0.006 |
| 49 | *Holophaga* | 1.59E-05 | 0.000318101 | 0.001 |
| 50 | *Inquilinus* | 0.000217073 | 9.07E-05 | 0.024 |
| 51 | *Janthinobacterium* | 0 | 0.000112223 | 0.007 |
| 52 | *Jonesia* | 0.000364863 | 0.000177101 | 0.022 |
| 53 | *Lacibacter* | 3.62E-05 | 0.000278803 | 0.003 |
| 54 | *Leifsonia* | 0.000599249 | 0.000140062 | 0.004 |
| 55 | *Lentibacillus* | 0.000160385 | 0 | 0.003 |
| 56 | *Leptolinea* | 0.000133519 | 0.000588909 | 0.033 |
| 57 | *Leucobacter* | 0.001590789 | 0.000197388 | 0.001 |
| 58 | *Litoribacter* | 0.000415029 | 6.03E-05 | 0.007 |
| 59 | *Longilinea* | 0.001572846 | 0.00347066 | 0.021 |
| 60 | *Lutibacter* | 7.95E-06 | 0.000109254 | 0.048 |
| 61 | *Lutispora* | 0.000263074 | 8.19E-05 | 0.015 |
| 62 | *Marinilactibacillus* | 0.000138407 | 0 | 0.007 |
| 63 | *Marinobacter* | 0.001201697 | 0.000166413 | 0.022 |
| 64 | *Massilia* | 7.95E-06 | 0.00016198 | 0.003 |
| 65 | *Methylocaldum* | 0.000248913 | 9.43E-05 | 0.034 |
| 66 | *Methylophilus* | 5.93E-05 | 0.000222089 | 0.03 |
| 67 | *Micrococcus* | 0.000595883 | 0.000231622 | 0.04 |
| 68 | *Millisia* | 0.000109893 | 0 | 0.04 |
| 69 | *Nitriliruptor* | 0.001240434 | 0.00038727 | 0.011 |
| 70 | *Ochrobactrum* | 0.000754082 | 0.000245794 | 0.002 |
| 71 | *Ornithinibacillus* | 8.37E-05 | 0 | 0.04 |
| 72 | *Ornithinimicrobium* | 0.000433374 | 0.00013654 | 0.003 |
| 73 | *Paenibacillus* | 0.002018443 | 0.000589064 | 0.041 |
| 74 | *Paenochrobactrum* | 0.000348968 | 0 | 0.022 |
| 75 | *Parapedobacter* | 0.001050208 | 0.000157601 | 0.007 |
| 76 | *Parvibaculum* | 0.000263808 | 6.91E-05 | 0.01 |
| 77 | *Phaeobacter* | 0.000464737 | 0.000150645 | 0.02 |
| 78 | *Pigmentiphaga* | 0.00030466 | 7.14E-05 | 0.013 |
| 79 | *Planococcaceae_incertae_sedis* | 0.000625782 | 0.000144608 | 0.011 |
| 80 | *Planococcus* | 0.000744091 | 6.78E-05 | 0.021 |
| 81 | *Pontibacter* | 0.004151875 | 0.001678583 | 0.023 |
| 82 | *Porphyrobacter* | 0.001104133 | 0.000515242 | 0.044 |
| 83 | *Prosthecobacter* | 0.000422317 | 0.001124921 | 0.027 |
| 84 | *Proteiniclasticum* | 0.010281141 | 0.00235731 | 0.023 |
| 85 | *Proteiniphilum* | 0.000800115 | 0.000262651 | 0.015 |
| 86 | *Pseudorhodoferax* | 0.000150644 | 1.63E-05 | 0.01 |
| 87 | *Pusillimonas* | 0.001502818 | 0.000397256 | 0.001 |
| 88 | *Runella* | 7.95E-06 | 0.00013042 | 0.013 |
| 89 | *Saccharofermentans* | 0.00369516 | 0.001739781 | 0.008 |
| 90 | *Saccharomonospora* | 0.000197867 | 1.89E-05 | 0.006 |
| 91 | *Salegentibacter* | 0.00011536 | 2.07E-05 | 0.029 |
| 92 | *Sandaracinobacter* | 4.71E-05 | 0.000258946 | 0.016 |
| 93 | *Sedimentibacter* | 0.001078412 | 0.000504036 | 0.011 |
| 94 | *Serpens* | 0.000583352 | 0.00013042 | 0.002 |
| 95 | *Solimonas* | 0.00015094 | 1.06E-05 | 0.017 |
| 96 | *Sphaerotilus* | 4.20E-05 | 0.000155411 | 0.013 |
| 97 | *Sphingobacterium* | 0.003736698 | 0.000786535 | 0.003 |
| 98 | *Sphingopyxis* | 0.001059063 | 0.000316557 | 0.024 |
| 99 | *Spirosoma* | 0 | 0.000154412 | 0.007 |
| 100 | *Sporichthya* | 4.22E-05 | 0.000150812 | 0.038 |
| 101 | *Sporolactobacillaceae_incertae_sedis* | 0.001004021 | 0.00020946 | 0.023 |
| 102 | *Sulfurovum* | 0.000216121 | 7.70E-05 | 0.04 |
| 103 | *Symbiobacterium* | 0.000265756 | 2.78E-05 | 0.002 |
| 104 | *Tepidimicrobium* | 7.54E-05 | 0 | 0.022 |
| 105 | *Thalassobacillus* | 0.000316269 | 0.000111777 | 0.033 |
| 106 | *Thermomonas* | 3.71E-05 | 0.000173965 | 0.024 |
| 107 | *Thiohalophilus* | 0.000235943 | 4.01E-05 | 0.004 |
| 108 | *Tissierella* | 0.003579311 | 0.000481825 | 0.001 |
| 109 | *Trichococcus* | 0.000597136 | 0.00022386 | 0.035 |
| 110 | *Truepera* | 0.002573534 | 0.001330641 | 0.004 |
| 111 | *Uliginosibacterium* | 7.95E-06 | 0.000125557 | 0.025 |
| 112 | *Vagococcus* | 0.000639466 | 0.000155284 | 0.008 |
| 113 | *Vasilyevaea* | 0.000372893 | 0.000149769 | 0.016 |
| 114 | *Victivallis* | 0.000132577 | 3.61E-05 | 0.031 |
| 115 | *Virgibacillus* | 0.000561843 | 0.000184003 | 0.043 |
| 116 | *Weissella* | 0.00063445 | 0.000207813 | 0.004 |
| 117 | *Wohlfahrtiimonas* | 0.00013007 | 0 | 0.022 |
| 118 | *Zavarzinella* | 0.003027712 | 0.006536424 | 0.049 |

Table S4: Significantly different bacteria at genus level between Dom and FecD solid wastes

| S/N | Features | Mean_Dom | Mean-FecD | P-value |
| --- | --- | --- | --- | --- |
| 1 | *Acetanaerobacterium* | 0.000746603 | 0.001422 | 0.012 |
| 2 | *Acetitomaculum* | 0.000120935 | 0.000392 | 0.008 |
| 3 | *Acidaminobacter* | 0.000104934 | 0.000199 | 0.041 |
| 4 | *Aciditerrimonas* | 0.002512702 | 0.001172 | 0.002 |
| 5 | *Acidocella* | 0 | 6.43E-05 | 0.033 |
| 6 | *Afifella* | 0.000134467 | 1.23E-05 | 0.021 |
| 7 | *Aggregatibacter* | 4.90E-05 | 0.000161 | 0.018 |
| 8 | *Alkanibacter* | 0 | 5.04E-05 | 0.033 |
| 9 | *Altererythrobacter* | 0.001015035 | 0.000605 | 0.028 |
| 10 | *Alterococcus* | 0.000293837 | 4.21E-05 | 0.003 |
| 11 | *Amaricoccus* | 0.000958262 | 0.000523 | 0.011 |
| 12 | *Anaerobacter* | 1.39E-05 | 0.000103 | 0.039 |
| 13 | *Anaerofustis* | 0.000199679 | 0.000559 | 0.003 |
| 14 | *Anaeromyxobacter* | 0.001668016 | 0.000599 | 0.001 |
| 15 | *Anaerovibrio* | 0.000217448 | 0.000634 | 0.016 |
| 16 | *Aquiflexum* | 0.000388661 | 0.00014 | 0.013 |
| 17 | *Arenimonas* | 0.000691665 | 0.000304 | 0.005 |
| 18 | *Armatimonadetes_gp5* | 0.001310721 | 0.000496 | 0.002 |
| 19 | *Atopobium* | 7.20E-06 | 0.00017 | 0.001 |
| 20 | *Azospirillum* | 0.000502205 | 0.000189 | 0.002 |
| 21 | *Azotobacter* | 0.000286 | 0.000136 | 0.017 |
| 22 | *Bacteriovorax* | 0.000416966 | 0.000134 | 0.011 |
| 23 | *Bauldia* | 0.000581718 | 0.00028 | 0.023 |
| 24 | *Bdellovibrio* | 0.001338809 | 0.000379 | 0.001 |
| 25 | *Bifidobacterium* | 0.000844653 | 0.001656 | 0.021 |
| 26 | *Blastopirellula* | 0.00479964 | 0.002606 | 0.02 |
| 27 | *Blautia* | 0.003389678 | 0.006994 | 0.007 |
| 28 | *Brachymonas* | 0.000363351 | 0.000106 | 0.002 |
| 29 | *BRC1_genera_incertae_sedis* | 0.001433748 | 0.000608 | 0.003 |
| 30 | *Bulleidia* | 0.000611672 | 0.001576 | 0.001 |
| 31 | *Butyricicoccus* | 0.000997864 | 0.001763 | 0.016 |
| 32 | *Byssovorax* | 0.00078541 | 0.000327 | 0.001 |
| 33 | *Caldilinea* | 0.004152518 | 0.001367 | 0.001 |
| 34 | *Campylobacter* | 0.000264356 | 0.000478 | 0.033 |
| 35 | *Cellulosilyticum* | 0.001626327 | 0.003434 | 0.013 |
| 36 | *Cesiribacter* | 0.000829334 | 0.00042 | 0.009 |
| 37 | *Chondromyces* | 0.001381393 | 0.000694 | 0.003 |
| 38 | *Cloacibacterium* | 0.000235321 | 0.000464 | 0.006 |
| 39 | *Clostridium_IV* | 0.005511419 | 0.010845 | 0.002 |
| 40 | *Clostridium_sensu_stricto* | 0.007506046 | 0.01936 | 0.002 |
| 41 | *Clostridium_XI* | 0.001954737 | 0.004209 | 0.007 |
| 42 | *Clostridium_XlVa* | 0.008145327 | 0.015203 | 0.001 |
| 43 | *Conexibacter* | 0.002212573 | 0.001257 | 0.008 |
| 44 | *Coprobacillus* | 0.000106587 | 0.000279 | 0.008 |
| 45 | *Coprococcus* | 0.001228479 | 0.00261 | 0.004 |
| 46 | *Cucumibacter* | 7.66E-05 | 0 | 0.012 |
| 47 | *Dasania* | 0.000126226 | 1.04E-05 | 0.013 |
| 48 | *Defluviicoccus* | 5.09E-05 | 9.65E-05 | 0.006 |
| 49 | *Desulfocapsa* | 0.000391447 | 9.77E-05 | 0.001 |
| 50 | *Desulfosarcina* | 0.000155236 | 3.22E-05 | 0.044 |
| 51 | *Dethiobacter* | 0.000373165 | 0.00015 | 0.019 |
| 52 | *Dorea* | 0.001250003 | 0.002564 | 0.003 |
| 53 | *Duganella* | 2.94E-05 | 0.00015 | 0.034 |
| 54 | *Dyella* | 6.19E-05 | 0.000178 | 0.027 |
| 55 | *Dysgonomonas* | 0.001469088 | 0.00053 | 0.011 |
| 56 | *Ensifer* | 0.000549336 | 0.000361 | 0.033 |
| 57 | *Enterorhabdus* | 0.000170542 | 0.000341 | 0.046 |
| 58 | *Erysipelotrichaceae_incertae_sedis* | 0.000686744 | 0.001463 | 0.002 |
| 59 | *Erythrobacter* | 0.000515678 | 0.00025 | 0.006 |
| 60 | *Ethanoligenens* | 0.000416154 | 0.000896 | 0.002 |
| 61 | *Eubacterium* | 0.001005086 | 0.001717 | 0.022 |
| 62 | *Euzebya* | 0.000722786 | 0.000184 | 0.002 |
| 63 | *Faecalibacterium* | 0.001553658 | 0.00286 | 0.011 |
| 64 | *Filomicrobium* | 0.000247885 | 7.81E-05 | 0.022 |
| 65 | *Flavihumibacter* | 0.000512247 | 0.00029 | 0.045 |
| 66 | *Flavobacterium* | 0.004622564 | 0.002613 | 0.003 |
| 67 | *Flavonifractor* | 0.000656434 | 0.001274 | 0.022 |
| 68 | *Fulvivirga* | 0.000381059 | 0.000154 | 0.008 |
| 69 | *Fusobacterium* | 0.000153858 | 0.000447 | 0.041 |
| 70 | *Geminicoccus* | 0.002238506 | 0.001408 | 0.028 |
| 71 | *Gemmatimonas* | 0.009127539 | 0.005655 | 0.04 |
| 72 | *Geobacter* | 0.000442748 | 0.000187 | 0.021 |
| 73 | *Gordonibacter* | 3.78E-05 | 0.000168 | 0.014 |
| 74 | *Gp10* | 0.001544591 | 0.000876 | 0.045 |
| 75 | *Gp16* | 0.003334035 | 0.002232 | 0.046 |
| 76 | *Gp4* | 0.008213745 | 0.004766 | 0.004 |
| 77 | *Gp7* | 0.00241369 | 0.001254 | 0.005 |
| 78 | *Guggenheimella* | 0.000497485 | 0.001003 | 0.031 |
| 79 | *Haliangium* | 0.000721728 | 0.00035 | 0.013 |
| 80 | *Haloferula* | 0.000381233 | 8.21E-05 | 0.001 |
| 81 | *Heliothrix* | 0.000793215 | 0.00018 | 0.002 |
| 82 | *Holdemania* | 0.00053518 | 0.00088 | 0.037 |
| 83 | *Holophaga* | 1.63E-05 | 7.06E-05 | 0.001 |
| 84 | *Howardella* | 0.00013478 | 0.000429 | 0.004 |
| 85 | *Ilumatobacter* | 0.000833083 | 0.000445 | 0.014 |
| 86 | *Isobaculum* | 3.11E-05 | 0.000159 | 0.046 |
| 87 | *Kineococcus* | 3.26E-06 | 7.49E-05 | 0.04 |
| 88 | *Klebsiella* | 1.60E-05 | 7.66E-05 | 0.036 |
| 89 | *Kofleria* | 0.000362884 | 0.000101 | 0.005 |
| 90 | *Lachnospiracea_incertae_sedis* | 0.004356635 | 0.008519 | 0.003 |
| 91 | *Lacibacter* | 1.74E-05 | 4.92E-05 | 0.016 |
| 92 | *Lentibacillus* | 0.000108757 | 0.000263 | 0.037 |
| 93 | *Levilinea* | 0.000652562 | 0.000154 | 0.001 |
| 94 | *Lewinella* | 0.000825868 | 0.000372 | 0.004 |
| 95 | *Marmoricola* | 0.00017075 | 2.27E-05 | 0.004 |
| 96 | *Meniscus* | 0.000584182 | 0.000236 | 0.04 |
| 97 | *Micromonospora* | 0.000275405 | 9.87E-05 | 0.002 |
| 98 | *Mitsuokella* | 0.000308978 | 0.000702 | 0.023 |
| 99 | *Mogibacterium* | 0.000718639 | 0.001509 | 0.013 |
| 100 | *Mucispirillum* | 5.72E-05 | 9.28E-05 | 0.036 |
| 101 | *Nannocystis* | 0.000352557 | 0.000205 | 0.042 |
| 102 | *Nitriliruptor* | 0.001411924 | 0.000451 | 0.003 |
| 103 | *Nitrosomonas* | 0.000331386 | 7.05E-05 | 0.002 |
| 104 | *Nitrospira* | 0.001108145 | 0.000416 | 0.005 |
| 105 | *Ochrobactrum* | 0.000405741 | 0.000698 | 0.008 |
| 106 | *Olivibacter* | 7.38E-05 | 0.000194 | 0.043 |
| 107 | *Orientia* | 2.71E-05 | 0.000239 | 0.003 |
| 108 | *Oscillibacter* | 0.003855594 | 0.006725 | 0.021 |
| 109 | *Oxalicibacterium* | 0.000348488 | 0.00061 | 0.045 |
| 110 | *Papillibacter* | 0.001719014 | 0.002684 | 0.045 |
| 111 | *Parapedobacter* | 0.000438052 | 0.000156 | 0.008 |
| 112 | *Pasteuria* | 0.00383163 | 0.002366 | 0.049 |
| 113 | *Pelotomaculum* | 0.000524176 | 0.0002 | 0.033 |
| 114 | *Peptoniphilus* | 0 | 0.000107 | 0 |
| 115 | *Peredibacter* | 0.001210559 | 0.000626 | 0.026 |
| 116 | *Perlucidibaca* | 5.72E-05 | 0.00035 | 0.037 |
| 117 | *Phaselicystis* | 0.000738131 | 0.000255 | 0.002 |
| 118 | *Phycisphaera* | 0.000813696 | 0.000234 | 0.002 |
| 119 | *Planctomyces* | 0.007419911 | 0.003534 | 0.003 |
| 120 | *Planobispora* | 0.000136178 | 1.65E-05 | 0.009 |
| 121 | *Plesiocystis* | 0.000281507 | 6.44E-05 | 0.003 |
| 122 | *Pontibacter* | 0.007202116 | 0.001755 | 0.011 |
| 123 | *Porticoccus* | 0.000234785 | 8.08E-05 | 0.006 |
| 124 | *Propionivibrio* | 9.66E-06 | 5.03E-05 | 0.003 |
| 125 | *Proteiniclasticum* | 0.003163074 | 0.001921 | 0.025 |
| 126 | *Proteiniphilum* | 0.000639268 | 0.000223 | 0.002 |
| 127 | *Pseudoflavonifractor* | 0.000498561 | 0.000876 | 0.042 |
| 128 | *Pseudofulvimonas* | 0.000614602 | 0.000331 | 0.03 |
| 129 | *Pseudoramibacter* | 0.000179478 | 0.000528 | 0.003 |
| 130 | *Pusillimonas* | 0.001228017 | 0.000612 | 0.026 |
| 131 | *Rhizobium* | 0.000861047 | 0.001309 | 0.04 |
| 132 | *Rhodococcus* | 0.000381501 | 0.000803 | 0.038 |
| 133 | *Robinsoniella* | 0.000234044 | 0.000509 | 0.018 |
| 134 | *Roseburia* | 0.000964684 | 0.002514 | 0.012 |
| 135 | *Rubrobacter* | 0.001167148 | 0.000649 | 0.029 |
| 136 | *Ruminococcus* | 0.005368898 | 0.009873 | 0.006 |
| 137 | *Rummeliibacillus* | 0.000157895 | 0.000496 | 0.01 |
| 138 | *Salegentibacter* | 0.000107375 | 0.000204 | 0.047 |
| 139 | *Salinibacter* | 0.000945083 | 0.000319 | 0.001 |
| 140 | *Salinimicrobium* | 0.000506092 | 0.000245 | 0.02 |
| 141 | *Salisaeta* | 0.001746535 | 0.000595 | 0.001 |
| 142 | *Sarcina* | 4.69E-05 | 0.000441 | 0.005 |
| 143 | *Sediminibacterium* | 9.20E-05 | 0.000292 | 0.01 |
| 144 | *Selenomonas* | 5.95E-05 | 0.000143 | 0.027 |
| 145 | *Sharpea* | 0.000163336 | 0.000363 | 0.021 |
| 146 | *Sinomonas* | 1.74E-05 | 0.000311 | 0.001 |
| 147 | *Skermanella* | 0.001206684 | 0.000659 | 0.012 |
| 148 | *Slackia* | 0.000136585 | 0.000383 | 0.002 |
| 149 | *Solitalea* | 0.00026293 | 5.15E-05 | 0.001 |
| 150 | *Sorangium* | 0.000309402 | 0.000144 | 0.047 |
| 151 | *Spartobacteria_genera_incertae_sedis* | 0.004300779 | 0.002713 | 0.022 |
| 152 | *Sphaerobacter* | 0.004549194 | 0.003089 | 0.021 |
| 153 | *Sphingosinicella* | 0.000970546 | 0.000519 | 0.006 |
| 154 | *Sporobacter* | 0.005496117 | 0.009297 | 0.007 |
| 155 | *Staphylococcus* | 0.000354923 | 0.000965 | 0.008 |
| 156 | *Steroidobacter* | 0.001434563 | 0.000919 | 0.008 |
| 157 | *Streptococcus* | 0.00049342 | 0.00144 | 0.002 |
| 158 | *Syntrophobacter* | 0.000456531 | 0.000157 | 0.007 |
| 159 | *Thermoleophilum* | 0.000923491 | 0.000504 | 0.026 |
| 160 | *Thermomicrobium* | 8.56E-05 | 6.21E-06 | 0.047 |
| 161 | *Thioalkalispira* | 0.000107837 | 1.04E-05 | 0.049 |
| 162 | *Thiohalobacter* | 0.000331364 | 0.000118 | 0.013 |
| 163 | *Thiohalomonas* | 0.000214997 | 7.73E-05 | 0.009 |
| 164 | *Thiohalophilus* | 0.000266669 | 3.41E-05 | 0.002 |
| 165 | *Thiomonas* | 0 | 7.44E-05 | 0.011 |
| 166 | *Truepera* | 0.002877661 | 0.001312 | 0.002 |
| 167 | *Tumebacillus* | 0.000487761 | 0.000188 | 0.005 |
| 168 | *Turicibacter* | 0.000908706 | 0.002883 | 0.002 |
| 169 | *Ulvibacter* | 0 | 6.28E-05 | 0.011 |
| 170 | *Vagococcus* | 0.00034104 | 0.000722 | 0.02 |
| 171 | *Vampirovibrio* | 0.002689868 | 0.004959 | 0.015 |
| 172 | *Veillonellaceae_genus_incertae_sedis* | 0 | 2.04E-05 | 0.033 |
| 173 | *Viridibacillus* | 0 | 8.05E-05 | 0.011 |

Table S5: Significantly different bacteria at genus level between Dom and Riv solid waste

| S/N | Features | Mean_Dom | Mean-Riv | P-value |
| --- | --- | --- | --- | --- |
| 1 | *Acetoanaerobium* | 0.00048 | 0.000222 | 0.013 |
| 2 | *Acholeplasma* | 0.001022 | 0.000321 | 0.037 |
| 3 | *Aequorivita* | 9.79E-05 | 0 | 0.042 |
| 4 | *Alishewanella* | 0.000265 | 0.000116 | 0.004 |
| 5 | *Alkaliphilus* | 0.000543 | 0.000146 | 0.015 |
| 6 | *Aminomonas* | 0.000116 | 2.07E-05 | 0.035 |
| 7 | *Anaerofilum* | 0.000156 | 4.01E-05 | 0.038 |
| 8 | *Anaeroplasma* | 0.000299 | 5.24E-05 | 0.027 |
| 9 | *Anderseniella* | 8.17E-05 | 0.000295 | 0.007 |
| 10 | *Armatimonadetes_gp5* | 0.001311 | 0.002515 | 0.033 |
| 11 | *Asanoa* | 2.38E-05 | 9.02E-05 | 0.016 |
| 12 | *Atopostipes* | 0.000525 | 0.000149 | 0.025 |
| 13 | *Azomonas* | 5.14E-05 | 0 | 0.045 |
| 14 | *Azotobacter* | 0.000286 | 0.000161 | 0.027 |
| 15 | *Bellilinea* | 0.003647 | 0.006184 | 0.045 |
| 16 | *Blastococcus* | 0.000864 | 0.000502 | 0.048 |
| 17 | *Brachybacterium* | 0.000297 | 0.000137 | 0.016 |
| 18 | *Brevibacillus* | 0.000222 | 6.22E-05 | 0.005 |
| 19 | *Brevibacterium* | 0.000451 | 0.000157 | 0.007 |
| 20 | *Brevundimonas* | 0.001523 | 0.00087 | 0.002 |
| 21 | *Caenispirillum* | 0.000132 | 0 | 0.006 |
| 22 | *Caldalkalibacillus* | 5.02E-05 | 0 | 0.042 |
| 23 | *Caldicoprobacter* | 0.000103 | 1.89E-05 | 0.023 |
| 24 | *Castellaniella* | 0.000469 | 0.000141 | 0.011 |
| 25 | *Caulobacter* | 9.49E-05 | 0.00032 | 0.042 |
| 26 | *Cerasicoccus* | 0.000386 | 0.000138 | 0.033 |
| 27 | *Cetobacterium* | 5.39E-06 | 5.63E-05 | 0.022 |
| 28 | *Clostridium_XII* | 9.12E-05 | 1.80E-05 | 0.035 |
| 29 | *Cohnella* | 0.000104 | 1.06E-05 | 0.035 |
| 30 | *Corynebacterium* | 0.001991 | 0.000735 | 0.002 |
| 31 | *Defluviicoccus* | 5.09E-05 | 0.000336 | 0.007 |
| 32 | *Desmospora* | 0.000323 | 9.64E-05 | 0.001 |
| 33 | *Desulfatirhabdium* | 1.45E-05 | 9.38E-05 | 0.04 |
| 34 | *Dethiobacter* | 0.000373 | 0.000105 | 0.023 |
| 35 | *Devosia* | 0.00214 | 0.001332 | 0.028 |
| 36 | *Dietzia* | 0.000651 | 0.000307 | 0.021 |
| 37 | *Duganella* | 2.94E-05 | 0.000123 | 0.024 |
| 38 | *Ensifer* | 0.000549 | 0.000342 | 0.037 |
| 39 | *Enterococcus* | 0.00075 | 0.000253 | 0.007 |
| 40 | *Erysipelothrix* | 0.000479 | 4.31E-05 | 0.002 |
| 41 | *Euzebya* | 0.000723 | 0.000222 | 0.01 |
| 42 | *Exiguobacterium* | 0.000603 | 0.00028 | 0.015 |
| 43 | *Ferruginibacter* | 0.000453 | 0.001499 | 0.012 |
| 44 | *Fervidicella* | 0.000119 | 0.00037 | 0.048 |
| 45 | *Fulvimonas* | 0 | 3.57E-05 | 0.021 |
| 46 | *Fulvivirga* | 0.000381 | 0.000135 | 0.012 |
| 47 | *Garciella* | 0.000447 | 4.23E-05 | 0.002 |
| 48 | *Gemmata* | 0.002323 | 0.004526 | 0.033 |
| 49 | *Geobacillus* | 0.000315 | 9.65E-05 | 0.01 |
| 50 | *Georgenia* | 0.000311 | 0.000175 | 0.049 |
| 51 | *Geothrix* | 3.01E-05 | 0.000168 | 0.04 |
| 52 | *Gp1* | 0.000306 | 0.00074 | 0.034 |
| 53 | *Gp17* | 0.000766 | 0.002473 | 0.011 |
| 54 | *Gp20* | 0 | 7.74E-05 | 0.006 |
| 55 | *Gp25* | 0.000222 | 0.001257 | 0.007 |
| 56 | *Hallella* | 1.56E-05 | 0.000239 | 0.04 |
| 57 | *Halochromatium* | 0 | 8.54E-05 | 0.002 |
| 58 | *Holdemania* | 0.000535 | 0.00027 | 0.034 |
| 59 | *Holophaga* | 1.63E-05 | 0.000318 | 0.001 |
| 60 | *Hyphomicrobium* | 0.001 | 0.002226 | 0.022 |
| 61 | *Ignatzschineria* | 0.000329 | 0.000118 | 0.036 |
| 62 | *Jonesia* | 0.000363 | 0.000177 | 0.018 |
| 63 | *Kerstersia* | 8.69E-05 | 0 | 0.042 |
| 64 | *Kocuria* | 0.000471 | 0.000218 | 0.035 |
| 65 | *Ktedonobacter* | 1.08E-05 | 7.28E-05 | 0.007 |
| 66 | *Labrys* | 2.24E-05 | 0.000151 | 0.002 |
| 67 | *Lacibacter* | 1.74E-05 | 0.000279 | 0.001 |
| 68 | *Lentibacillus* | 0.000109 | 0 | 0.009 |
| 69 | *Leucobacter* | 0.000718 | 0.000197 | 0.002 |
| 70 | *Leuconostoc* | 0.000818 | 0.000264 | 0.018 |
| 71 | *Litoribacter* | 0.000318 | 6.03E-05 | 0.033 |
| 72 | *Longilinea* | 0.001674 | 0.003471 | 0.018 |
| 73 | *Lutibacter* | 2.15E-05 | 0.000109 | 0.031 |
| 74 | *Lutispora* | 0.000255 | 8.19E-05 | 0.047 |
| 75 | *Marichromatium* | 0 | 6.65E-05 | 0.006 |
| 76 | *Marinilactibacillus* | 8.62E-05 | 0 | 0.042 |
| 77 | *Marinobacter* | 0.000579 | 0.000166 | 0.023 |
| 78 | *Meiothermus* | 5.01E-06 | 6.03E-05 | 0.022 |
| 79 | *Methylomonas* | 3.77E-05 | 0.000131 | 0.038 |
| 80 | *Methylophilus* | 3.39E-05 | 0.000222 | 0.012 |
| 81 | *Methylopila* | 0 | 4.14E-05 | 0.021 |
| 82 | *Micromonospora* | 2.75E-04 | 0.00011 | 0.012 |
| 83 | *Nakamurella* | 2.59E-05 | 8.54E-05 | 0.04 |
| 84 | *Nesterenkonia* | 0.000322 | 5.81E-05 | 0.04 |
| 85 | *Nitriliruptor* | 0.001412 | 0.000387 | 0.006 |
| 86 | *Ochrobactrum* | 0.000406 | 0.000246 | 0.03 |
| 87 | *Ohtaekwangia* | 0.002061 | 0.003737 | 0.006 |
| 88 | *Ornithinimicrobium* | 0.000311 | 0.000137 | 0.006 |
| 89 | *Paenibacillus* | 0.001424 | 0.000589 | 0.019 |
| 90 | *Paenisporosarcina* | 0.000195 | 0 | 0.001 |
| 91 | *Paenochrobactrum* | 0.000161 | 0 | 0.003 |
| 92 | *Parapedobacter* | 0.000438 | 0.000158 | 0.025 |
| 93 | *Pasteuria* | 0.003832 | 0.007981 | 0.04 |
| 94 | *Phaeobacter* | 0.000384 | 0.000151 | 0.004 |
| 95 | *Planococcus* | 0.000428 | 6.78E-05 | 0.025 |
| 96 | *Planomicrobium* | 0.000121 | 0 | 0.026 |
| 97 | *Pontibacter* | 0.007202 | 0.001679 | 0.012 |
| 98 | *Propionivibrio* | 9.66E-06 | 0.00028 | 0.002 |
| 99 | *Prosthecobacter* | 0.00055 | 0.001125 | 0.034 |
| 100 | *Proteiniborus* | 7.57E-05 | 0 | 0.042 |
| 101 | *Proteiniphilum* | 0.000639 | 0.000263 | 0.032 |
| 102 | *Pusillimonas* | 0.001228 | 0.000397 | 0.001 |
| 103 | *Riemerella* | 0.000646 | 0.000319 | 0.017 |
| 104 | *Rivibacter* | 0 | 4.75E-05 | 0.021 |
| 105 | *Runella* | 1.66E-05 | 0.00013 | 0.001 |
| 106 | *Saccharomonospora* | 0.000171 | 1.89E-05 | 0.009 |
| 107 | *Salisaeta* | 1.75E-03 | 0.000994 | 0.03 |
| 108 | *Sandaracinobacter* | 0.000114 | 0.000259 | 0.025 |
| 109 | *Schlesneria* | 3.26E-06 | 5.07E-05 | 0.022 |
| 110 | *Sediminibacterium* | 9.20E-05 | 0.000443 | 0.012 |
| 111 | *Serpens* | 0.000313 | 0.00013 | 0.006 |
| 112 | *Shinella* | 0.000325 | 0.000197 | 0.045 |
| 113 | *Silanimonas* | 3.32E-06 | 8.54E-05 | 0.007 |
| 114 | *Skermanella* | 0.001207 | 0.000654 | 0.033 |
| 115 | *Soehngenia* | 0.000169 | 3.96E-05 | 0.038 |
| 116 | *Sphaerobacter* | 0.004549 | 0.002999 | 0.034 |
| 117 | *Sphingobacterium* | 0.002833 | 0.000787 | 0.002 |
| 118 | *Spirosoma* | 3.26E-06 | 0.000154 | 0.002 |
| 119 | *Sporichthya* | 2.87E-05 | 0.000151 | 0.002 |
| 120 | *Sporolactobacillaceae_incertae_sedis* | 0.000467 | 0.000209 | 0.021 |
| 121 | *Subdoligranulum* | 0.000586 | 0.000231 | 0.042 |
| 122 | *Sulfuritalea* | 0 | 4.81E-05 | 0.006 |
| 123 | *Sulfurovum* | 0.000232 | 7.70E-05 | 0.032 |
| 124 | *Symbiobacterium* | 0.000195 | 2.78E-05 | 0.003 |
| 125 | *Syntrophaceticus* | 0.000128 | 1.89E-05 | 0.006 |
| 126 | *Tepidanaerobacter* | 0.00036 | 1.73E-05 | 0.001 |
| 127 | *Tepidibacter* | 1.05E-05 | 8.50E-05 | 0.019 |
| 128 | *Tepidimicrobium* | 0.000108 | 0 | 0 |
| 129 | *Thalassobacillus* | 0.000239 | 0.000112 | 0.025 |
| 130 | *Thermoactinomyces* | 0.000483 | 0.000191 | 0.045 |
| 131 | *Thiobacter* | 9.35E-06 | 9.60E-05 | 0.016 |
| 132 | *Thiohalomonas* | 0.000215 | 8.09E-05 | 0.039 |
| 133 | *Thiohalophilus* | 0.000267 | 4.01E-05 | 0.005 |
| 134 | *Tissierella* | 0.001648 | 0.000482 | 0.003 |
| 135 | *Truepera* | 0.002878 | 0.001331 | 0.004 |
| 136 | *Uliginosibacterium* | 1.24E-05 | 0.000126 | 0.002 |
| 137 | *Ureibacillus* | 0.000247 | 2.95E-05 | 0.017 |
| 138 | *Vasilyevaea* | 0.000265 | 0.00015 | 0.033 |
| 139 | *Veillonellaceae_genus_incertae_sedis* | 0 | 4.51E-05 | 0.021 |
| 140 | *Victivallis* | 0.000151 | 3.61E-05 | 0.038 |
| 141 | *Weissella* | 0.001025 | 0.000208 | 0.004 |
| 142 | *Wohlfahrtiimonas* | 0.000154 | 0 | 0.006 |
| 143 | *Zavarzinella* | 0.002096 | 0.006536 | 0.013 |
|  |  |  |  |  |
