# Supplementary material for: High-throughput sequencing of 16S rRNA Gene Reveals Substantial Bacterial Diversity on the Municipal Dumpsite
Source: BMC Microbiol. 2016 Jul 11;16:145. doi: 10.1186/s12866-016-0758-8 (PMC4940873; doi:10.1186/s12866-016-0758-8)
Supplement: Additional file 5: — OTU based dendrogram demonstrating the similarity and differences of bacteria communities’ structure and membership from different solid wastes. Results were obtained using the Yue & Clayton measure (Fig a) and the Jaccard index (Fig b). (DOCX 1469 kb) [file 12866_2016_758_MOESM5_ESM.docx]

OTU based dendrogram demonstrating the similarity and differences of bacteria communities’ structure and membership from different solid wastes.


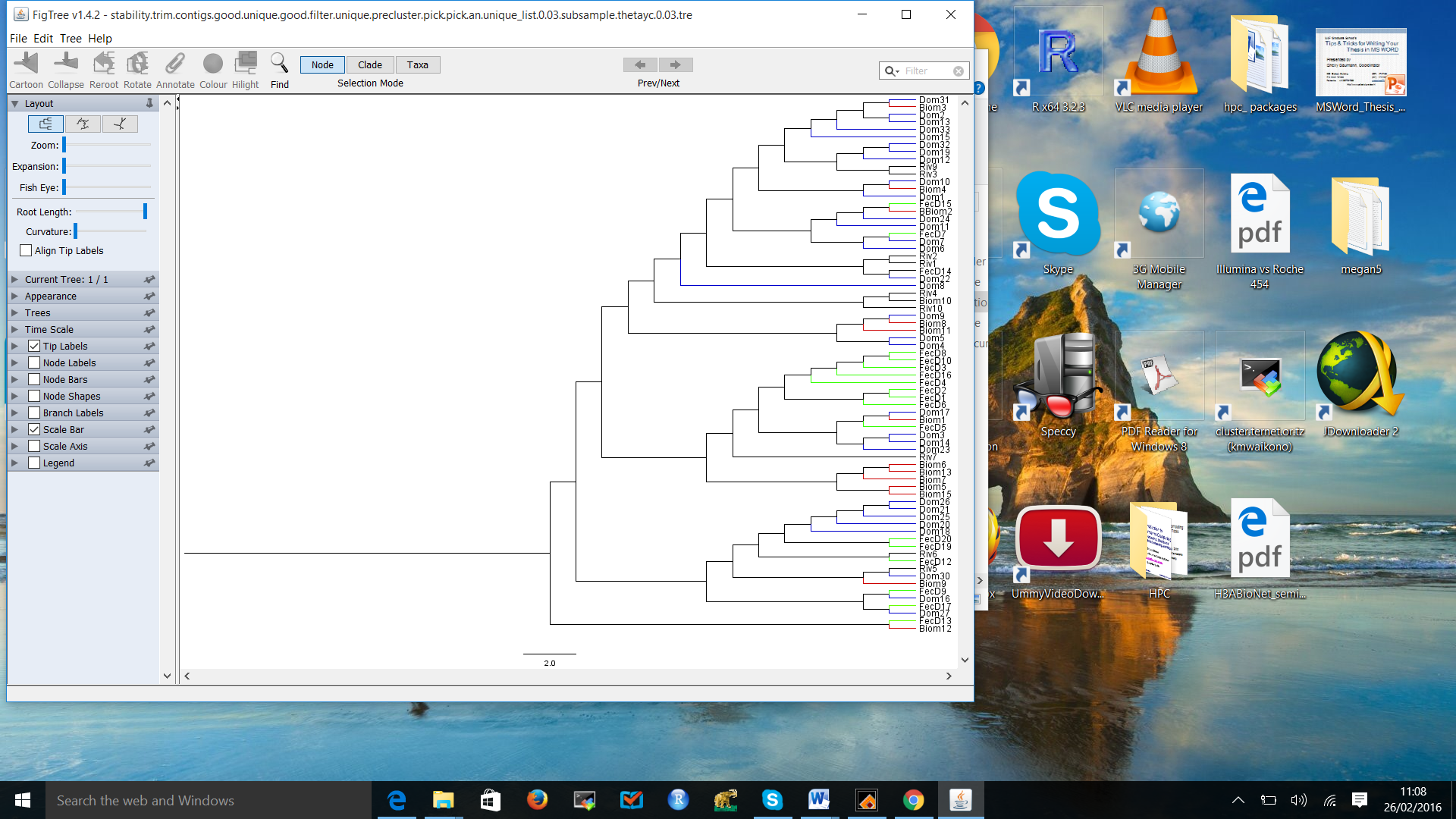


Figure S1 (a): OTU based dendrogram demonstrating the similarity and differences of the bacterial community structure from different types of solid waste. The dendrogram is based on Yue & Clayton indices.


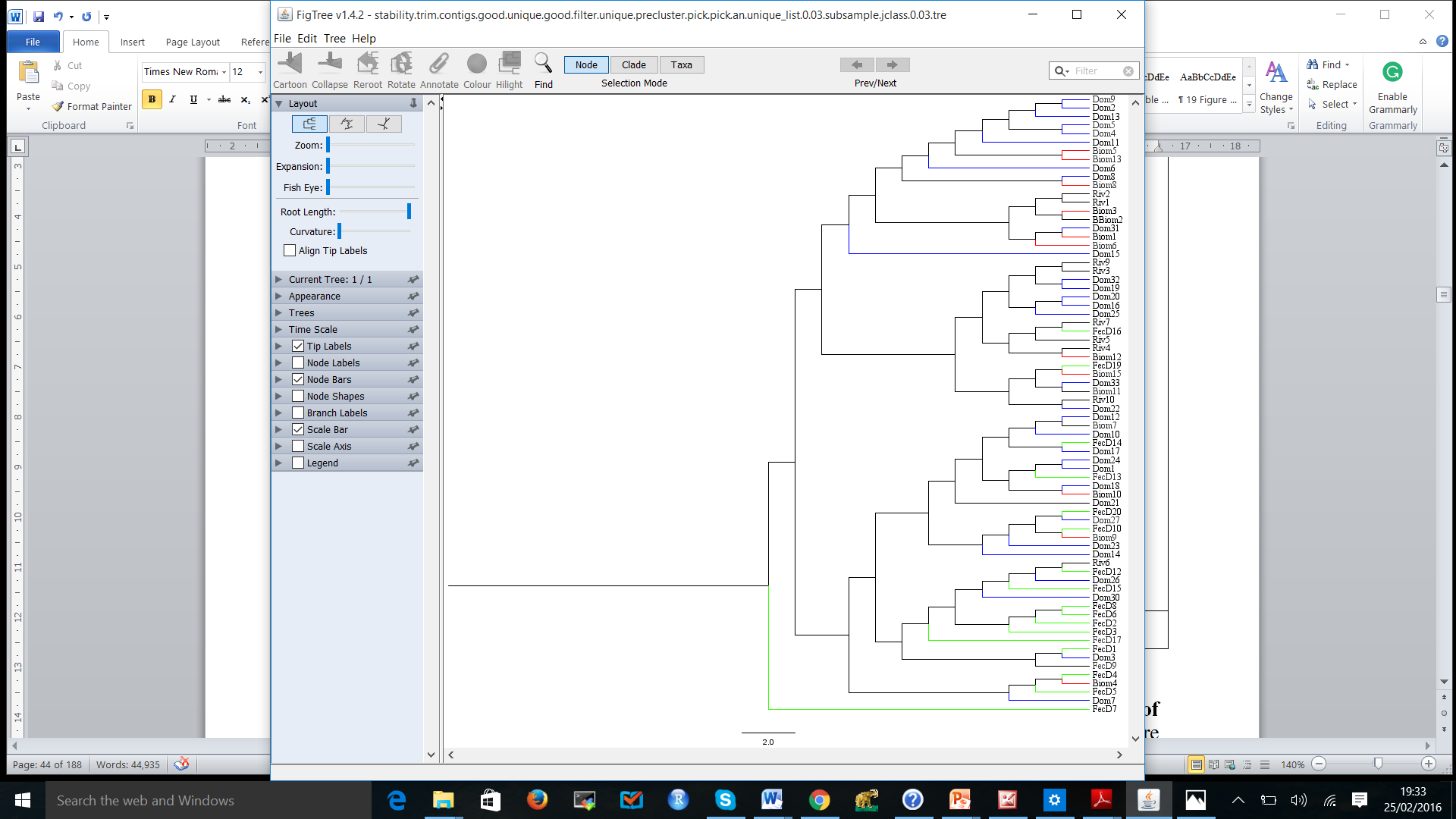
 Figure S1 (b): OTU based dendrogram demonstrating the similarity and differences of the bacterial community membership from different solid wastes. The dendrogram is based on traditional Jaccard index
